# Synergistic zeolite synthesis *via* a fluoride-deficient mixed approach

**DOI:** 10.1039/d5sc04097c

**Published:** 2025-10-13

**Authors:** Xuechao Tan, Miguel A. Camblor, Suk Bong Hong

**Affiliations:** a Center for Ordered Nanoporous Materials Synthesis, Division of Environmental Science and Engineering, POSTECH Pohang 37673 Korea sbhong@postech.ac.kr; b Instituto de Ciencia de Materiales de Madrid (ICMM), CSIC Madrid 28049 Spain

## Abstract

Here, we present a new fluoride-deficient synthesis approach to beta nanozeolites that results in synergistic effects of mineralizers on the crystallization kinetics, attained at intermediate fluoride and hydroxide concentrations (controlled by the ratio of hydrofluoric acid (HF) to organic structure-directing agent (OSDA) in hydroxide form). This approach represents an improved methodology as compared to the traditional hydroxide and fluoride routes, and it is likely transferable to other zeolites with different framework topologies. The method affords not only improved kinetics but also a control of the crystal size, reaching the nanoscale regime (≤100 nm) and framework defect concentration (≤3.5 per unit cell). Upon loading with Pd species, the resulting Pd/beta catalysts are active for wet methane combustion and their performance is dependent on the concentration of zeolite defects, crystal size, and zeolite Si/Al ratio. The 3.0 wt% Pd catalyst supported on Na^+^-post-exchanged 500 °C-calcined beta zeolite with a Si/Al ratio of 45 and a crystal size of *ca.* 75 nm, obtained at a synergistic mixture HF/OSDA ratio of 0.50, is the best among those studied: it shows a light-off temperature of 300 °C, together with excellent catalyst stability (over 90% methane conversion at 350 °C even after 100 h on stream), in the presence of 10% water vapor. This study provides an example where the performance of zeolite-supported metal catalysts can be significantly improved by controlling both the zeolite crystal size and defect concentration.

## Abstract 2

A fluoride-deficient synthesis approach to beta zeolites was developed to control the crystallization kinetics, crystal size and framework defect concentration, allowing beta-supported Pd catalysts to have better performance for wet CH_4_ combustion.

## Graphical abstract

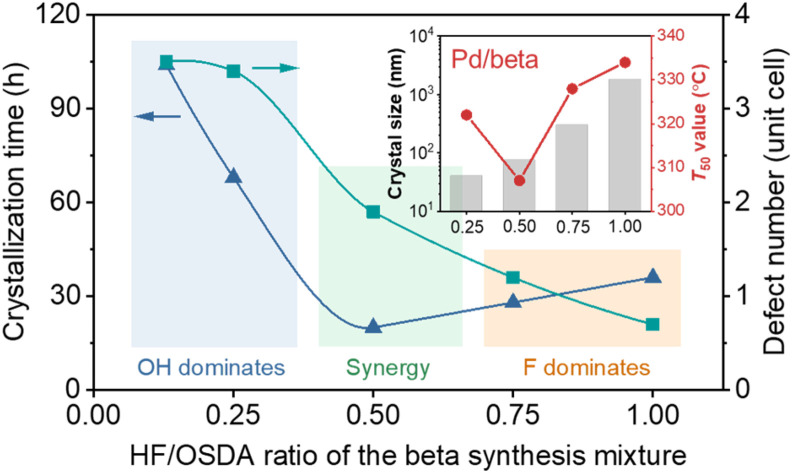

## 1. Introduction

The synthesis of zeolites has traditionally relied on two distinct approaches according to the “mineralizer” used: the hydroxide (OH^−^) route and the fluoride (F^−^) one. A mineralizer is a catalyst for the breaking and formation of Si–O–T bonds, where T is Si, Al, or other tetrahedral atom building up the zeolite framework. Each synthesis method offers unique advantages and limitations in terms of framework control, crystal size and morphology. However, no attempts to harmonize the mineralizing abilities of OH^−^ and F^−^ anions have been made so far. A notable exception is the very recent paper by Yu *et al.*,^1^ in which the addition of some HF to an otherwise OH^−^-mediated synthesis is shown to both improve the reproducibility and shorten the crystallization time of the new extra-large pore zeolite ZEO-1.^2^

Compared to conventional micrometer-sized zeolites, nanometer-sized zeolites (nanozeolites, ≤100 nm) have a distinct advantage as catalysts and catalyst supports because shorter diffusion path lengths and larger external surface areas mitigate mass transport limitations and improve diffusion of reactants and products. In principle, this maximizes the activity and reduces overreaction, improving selectivity and minimizing pore blockage. Therefore, considerable effort has been devoted to the synthesis of nanozeolites with different framework structures and compositions.^3–5^ However, zeolite structures that can be synthesized as nanozeolites are less than 10% of all zeolite framework types approved by the Structure Commission of the International Zeolite Association (260).^6–8^ Therefore, the synthesis of nanozeolites remains challenging.

In general, nanozeolites are synthesized under hydrothermal conditions at relatively low temperatures in hydroxide media to favor nucleation over crystal growth.^4,5^ Consequently, little attention has been devoted to the synthesis of nanozeolites in fluoride media, which generally takes place at near-neutral or slightly alkaline pH: the lower supersaturation compared to the hydroxide synthesis route typically results in the formation of relatively large zeolite crystals with a significantly lower concentration of connectivity defects.^9,10^ Although there are some reports on nanozeolites synthesized in the presence of F^−^ ions,^11–13^ virtually no attention has been directed toward the reasons for the nanometer size, which is likely related to the particular OSDA used. On the other hand, internal silanol (SiOH) defects in high-silica zeolites frequently cause coking, stability loss and low selectivity in acid-catalyzed hydrocarbon reactions,^14^ but they can serve as nucleation sites for the formation of small metal nanoparticles, resulting in highly robust catalysts for many important industrial processes.^15^

Over the past decade, our group has developed new synthetic methodologies that include the synergistic use of OSDAs and inorganic structure-directing agents (ISDAs) in the search for novel zeolite structures and compositions.^16^ Among them, the excess fluoride approach, aimed to induce changes in the zeolite phase selectivity by increasing the HF/OSDA ratio from 1.0 up to 4.0 in the presence of unselective OSDAs, allowed us to discover several new zeolite structures (PWO, PWW, PTY and PTF), as well as one new intergrowth family of pure-silica zeolites (*i.e.* PST-24).^17–20^ This approach is based on the hypothesis that the nature and extent of interactions during the zeolite nucleation process between the OSDA and silicate species could be significantly influenced by increasing F^−^ concentration in the starting mixture, thereby leading to a previously unobserved phase. We considered it was also worth investigating the effects of an F^−^ concentration decrease on the framework structure and/or physicochemical properties of the zeolite formed because in a fluoride-depleted synthesis, two mineralizers (OH^−^ and F^−^) could come into play. However, to our knowledge, little attention has been paid to this topic, especially with respect to the crystallization kinetics and the formation of internal framework defects in aluminosilicate zeolites.

Beta zeolite is a large-pore intergrowth of several polymorphs^6^ and is one of the five most produced zeolitic materials owing to its wide use as a commercial catalyst in hydrocracking, alkylation and acylation.^21^ Camblor and co-workers were the first to report beta nanozeolites with Si/Al ratios of 6.5–49 and crystal sizes of 10–100 nm using tetraethylammonium hydroxide (TEAOH) as an organic-structure-directing agent (OSDA) in alkali cation-free hydroxide media.^22,23^ They also reported the synthesis of beta zeolites with Si/Al ratios of 7.2–∞ in fluoride media, but the crystals obtained are microcrystalline in nature (0.5–5.0 μm, depending on their Si/Al ratio).^24^ For both types of syntheses, the crystallization times were exceedingly long, especially for the highest Al contents and smallest crystal sizes.

The present study describes a novel “fluoride-deficient” mixed approach that bridges these conventional methods, revealing an unexpected synergistic effect in zeolite formation. By carefully balancing the OH^−^ and F^−^ components, we have identified a compositional sweet spot where the interplay between these mineralizing agents leads to enhanced zeolite crystallization, increasing the crystallization rate beyond those found in the pure hydroxide and fluoride approaches and decreasing the crystal size down to the nanometer scale. Here we show that the average size of discrete beta crystals decreases dramatically from 1000–2500 to 30–250 nm when the mixture HF/OSDA and Si/Al ratios decrease and increase from 1.0 to 0.13 and from 12.5 to 200, respectively. Thus, the fluoride-deficient approach allows the synthesis of beta nanocrystals in an expeditious way. We also show that the fluoride-deficient approach generates a considerable amount of internal siloxy (SiO^−^) groups in as-made aluminosilicate beta nanozeolites.

On the other hand, methane (CH_4_), the main component of natural gas, is an excellent carbon-based fuel because of its high energy content, clean combustion products and low carbon dioxide (CO_2_) emission.^25^ In fact, while natural gas engines already have a huge market in lean-burn vehicles and maritime applications, the global warming potential of CH_4_ is 25 times higher than that of CO_2._^26,27^ Thus, the complete combustion of unburnt CH_4_, especially at low temperatures (300–400 °C), in natural gas engine exhausts is of great environmental concern.^28–32^ This has led us to introduce Pd, the most active CH_4_ combustion catalyst studied so far, into a series of beta zeolites with different Si/Al ratios synthesized here and to compare their catalytic performance for wet CH_4_ combustion.

## 2. Experimental

### 2.1 Beta zeolite synthesis and catalyst preparation

The synthesis of beta zeolites with different Si/Al ratios (14 – ∞) in fluoride media was performed by a modification of the procedures developed by Camblor *et al.*^24^ In a typical synthesis, tetraethylorthosilicate (TEOS, 98%, Aldrich) was hydrolyzed in an aqueous solution of TEAOH (35 wt%, Aldrich). Another solution, when used, was prepared by dissolving an appropriate amount of Al metal powder (99.5%, Sachem) in aqueous TEAOH. These two solutions were then mixed and stirred until the ethanol formed upon TEOS hydrolysis was completely evaporated. Finally, a given amount of hydrofluoric acid (HF, 48 wt%, Fisher) was added. The composition of the resulting mixture was 45.0TEAOH·*x*HF·*y*HAl_2_O_3_·80.0SiO_2_·720H_2_O, where *x* and *y* are varied between 5.8 ≤ *x* ≤ 45.0 and 0 ≤ *y* ≤ 3.2, respectively. The synthesis mixture was then charged into Teflon-lined 45-mL autoclaves and heated with rotation (60 rpm) at 140 °C for 0.1–28 days. The solid products were recovered by filtration followed by washing with deionized water or by repeated centrifugation (15 000 rpm, 10 min). The majority of starting mixtures and final products were found to have no liquid phase; therefore, the relative pH was measured after adding 1.0 g of starting mixtures or final products into 10 mL of deionized water, with the resulting slurry being stirred at room temperature for 0.5 h. The yield of each solid product was calculated in terms of the fractions of Al_2_O_3_ and SiO_2_ converted from the synthesis mixture into the solid product.

As-made zeolites were calcined in air at 550 °C for 8 h and refluxed twice in 1.0 M NH_4_NO_3_ solution (100 mL solution per 1.0 g zeolite) for 6 h to obtain the ammonium form. For catalytic comparison, NH_4_-beta with Si/Al = 12.5 (CP814E) was obtained from Zeolyst. Pd ion exchange was carried out by refluxing the NH_4_-form of beta zeolites in 1.5–3.0 × 10^−3^ M Pd(NO_3_)_2_·2H_2_O (40% Pd, Aldrich) solutions (1 g solid per 100 mL solution) with pH around 3.0 (adjusted by HNO_3_ solution) at 80 °C for 24 h. After filtering and drying at 120 °C for 12 h, Pd^2+^-exchanged beta zeolites were calcined in air at 500 °C for 5 h. If necessary, Na^+^ ions were introduced to eliminate the residual Brønsted acid sites (BASs) by refluxing twice calcined Pd/beta in 1.0 M NaNO_3_ solutions (100 mL solution per 1 g solid) at 80 °C for 6 h, and again calcined in air at 500 °C for 3 h. Here, we denote Pd beta zeolites as *x*Pd/M-beta-*m-n*, where *x*, M, *m* and *n* indicate the weight percent (wt%) of Pd, the type of extra-framework cation (H^+^ or Na^+^), the Si/Al and HF/OSDA ratios of beta synthesis mixtures, respectively.

### 2.2 Catalysis

Wet CH_4_ combustion was performed under atmospheric pressure in a packed-bed flow reactor.^33^ Prior to each catalytic run, 0.3 g of catalyst was pelleted to 20/30 mesh size, packed into a 3/8ʺ quartz tube reactor and activated in air flow at 500 °C for 0.5 h. The inlet and outlet gas temperatures were measured using two thermocouples placed above and below the catalyst, respectively. Then, a gas mixture consisting of 1500 ppm CH_4_, 5% O_2_ and 10% H_2_O in N_2_ balance at a gas hourly space velocity (GHSV) of 100 000 h^−1^ was fed into the reactor system. The CH_4_ conversion was measured in the temperature range of 200–500 °C at a temperature interval of 20 °C. The inlet and outlet CH_4_ and CO_2_ concentrations were analyzed online using a Thermo Nicolet 6700 FT-IR spectrometer with a 2 m gas cell. The long-term stability test was also performed under wet conditions (10% water vapor in the feed gas) at 350 °C for 100 h, where the partial CH_4_ oxidation was insignificant. Kinetic experiments were carried out by controlling the catalyst amount to make sure CH_4_ conversions were lower than 20%. The reaction rate for CH_4_ oxidation was calculated using the same equation as that used in our recent paper.^33^Reaction rate (s^−1^) = [(*C* × *X* × *v*)/(ML/MW)]where *C*, *X* and *v* are CH_4_ concentration (mol L^−1^) in feed gas, CH_4_ conversion (%) and volumetric flow rate (L s^−1^), respectively. ML and MW represent Pd loading (g) and Pd molecular weight (106.42 g mol^−1^), respectively. The apparent activation energy (*E*_app_) was determined from the slope of the Arrhenius plot.

### 2.3 Characterization

Powder X-ray diffraction (PXRD) patterns were measured on a PANalytical X'Pert diffractometer (Cu Kα radiation) with an X'Celerator detector. The crystallinity of all beta zeolites synthesized here was determined by comparing the area of the most intense X-ray peak at 2*θ* = 24.2° with that of pure-silica beta zeolite obtained under standard fluoride conditions (HF/OSDA = 1.00). Elemental analysis was carried out on a Jarrell-Ash Polyscan 61E inductively coupled plasma spectrometer in combination with a PerkinElmer 5000 atomic absorption spectrophotometer by POMIA (Pohang Institute of Metal Industry Advancement) Laboratories. Crystal morphology and size of beta zeolites and particle size of PdO were identified using a HITACHI S-4800 field-emission scanning electron microscope (FE-SEM), a JEOL JSM-6510 scanning electron microscope (SEM) and/or a JEOL JEM-2010 transmission electron microscope (TEM). Thermogravimetric analyses (TGA) were performed in air on an SII EXSTAR 6000 thermal analyzer, where weight loss related to the combustion of OSDA was determined from differential thermal analyses (DTA) using the same analyzer.

The N_2_ sorption data were obtained at −196 °C on a Mirae SI nanoPorosity-XG analyzer. Prior to each test, the sample (0.1 g) was pretreated at 300 °C for 3 h under vacuum. The micropore and mesopore volumes were calculated by *t*-Plot and BJH methods, respectively. A *P*/*P*_0_ range of 0.05–0.2 was used for the BET equation. The IR spectra in the framework and OH vibration regions were recorded on a Thermo-Nicolet 6700 FT-IR spectrometer equipped with an MCT detector, using KBr and self-supporting zeolite wafers (*ca.* 20 mg), respectively. The self-supporting wafer was pretreated in a home-built IR cell under vacuum (10^−4^ Pa) at 450 °C for 2 h. The Raman spectra were recorded on a Bruker FRA106/S FT-Raman spectrometer equipped with an Nd:YAG laser operating at 1064 nm. A 5-mm tube was used as the sample holder for zeolite powder, and the sample was exposed to a laser power of 100–250 mW at a spectral resolution of 2 cm^−1^. Typically, *ca.* 2000 scans were accumulated.

The ^27^Al and ^29^Si MAS spectra at a spinning speed of 21.0 kHz were recorded on a Bruker AVANCE III 500 spectrometer at ^27^Al and ^29^Si frequencies of 130.351 and 99.357 MHz, π/6 and π/2 rad pulse lengths of 1.0 and 4.0 μs, recycle delays of 2.0 and 40 s and acquisitions of *ca.* 100 and 500 pulse transients, respectively. The ^27^Al chemical shifts are reported relative to an Al(H_2_O)_6_^3+^ solution. The ^1^H MAS NMR spectra at a spinning speed of 10.0 kHz were collected at a ^1^H frequency of 500.57 MHz, a π/2 rad pulse length of 4.2 μs, a recycle delay of 4.0 s and an acquisition of about 32 pulse transients. The ^29^Si and ^1^H chemical shifts are reported relative to TMS. The ^19^F MAS NMR spectra at spinning speeds of 10.0 and 15.0 kHz were recorded at a ^19^F frequency of 470.527 MHz, a π/4 rad pulse length of 4.0 μs, a recycle delay of 5.0 s and an acquisition of about 6000 pulse transients. To determine the F^−^ content in each of the as-made beta zeolites, the same amount of sample was used in ^19^F MAS NMR experiments. Intensity normalization was made against the spectrum of pure-silica beta synthesized under standard fluoride conditions. The ^19^F chemical shifts are referenced relative to CFCl_3_.

NH_3_ temperature-programmed desorption (TPD) profiles were obtained from 150 to 700 °C on a fixed-bed, flow-type apparatus equipped with a thermal conductivity detector. O_2_ TPD data were collected on a Micromeritics AutoChem II 2920 analyzer. The same amount (0.1 g) of each catalyst was used in the measurement to accurately compare their signal intensity. After being pretreated (5% O_2_ in He, 50 mL min^−1^) at 300 °C for 2 h and cooled down to 50 °C, the TPD data were recorded up to 900 °C at a ramping rate of 10 °C min^−1^. Pulsed CO chemisorption was operated on the same equipment following the procedures described elsewhere.^33^ The UV-visible diffuse reflectance (UV-Vis DR) spectra were acquired on a Shimadzu UV-2501 PC spectrophotometer using BaSO_4_ as a reference. The X-ray absorption near-edge structure (XANES) and extended X-ray absorption fine structure (EXAFS) spectra at the Pd *K*-edge were recorded on the 10 C beamline at the PAL (Pohang, Korea) using a Si (111) crystal monochromator and analyzed using the Demeter software package (Version 0.9.26).^34^ The *k*^3^-weighted EXAFS Fourier transformation was carried out in the *k* range of 3.0–12.5 Å^−1^. A *S*_0_^2^ value of 0.75 was obtained from fitting the spectra of Pd foil and PdO (99.97%, Aldrich), and the same *S*_0_^2^ value was used in the other spectral fitting.

The approximate number of SiO^−^⋯HOSi defects per unit cell (64 T-atoms) of as-made beta zeolites synthesized here was calculated as follows:*N*_defect_ = *N*_OSDA_ – (*N*_Al_ + *N*_F_)where *N*_defect_, *N*_OSDA_, *N*_Al_ and *N*_F_ are the numbers of SiO^−^ defects, TEA^+^ cations, framework Al atoms and encapsulated F^−^ ions per unit cell, respectively. *N*_OSDA_ and *N*_Al_ were determined by thermal and elemental analyses, respectively. *N*_F_ was determined by comparing the total area of ^19^F resonances with that of beta-∞-1.00 (pure-silica beta zeolite synthesized at HF/OSDA = 1.00) that was assumed to have six F^−^ ions per unit cell.

## 3. Results and discussion

### 3.1 Zeolite synthesis and general characterization

Very recently, we have reported that the framework structure of zeolite supports and their composition (*i.e.* framework Si/Al ratio and extra-framework cation type) are crucial factors affecting the low-temperature activity and stability of zeolite-supported Pd catalysts for wet CH_4_ combustion.^33^ Among more than 50 catalysts studied, a 3.0 wt% Pd catalyst supported on Na^+^-post-exchanged 500 °C-calcined ITQ-27 (IWV) zeolite with a Si/Al ratio of 45, a large-pore zeolite with two intersecting 12-ring channels,^35^ was the best in terms of light-off temperature (temperature at 50% CH_4_ conversion), *i.e. T*_50_ (290 °C), and long-term catalyst stability (85% methane conversion after 100 h on stream at 330 °C in the presence of 10% water vapor). We also found that beta zeolite is the second-best support structure for Pd. On the other hand, it has long been recognized that increasing Al content in the beta synthesis mixture under both hydroxide and fluoride conditions requires longer crystallization times.^22,24^ Therefore, we performed zeolite synthesis with rotation (60 rpm) at 140 °C under fluoride-deficient conditions to check whether synthesis mixtures with a fixed Si/Al ratio (40) but different HF/OSDA ratios (0.13–1.00) yielded pure beta zeolite in the presence of TEAOH as an OSDA, and we also followed their crystallization kinetics.

**Fig. 1:**
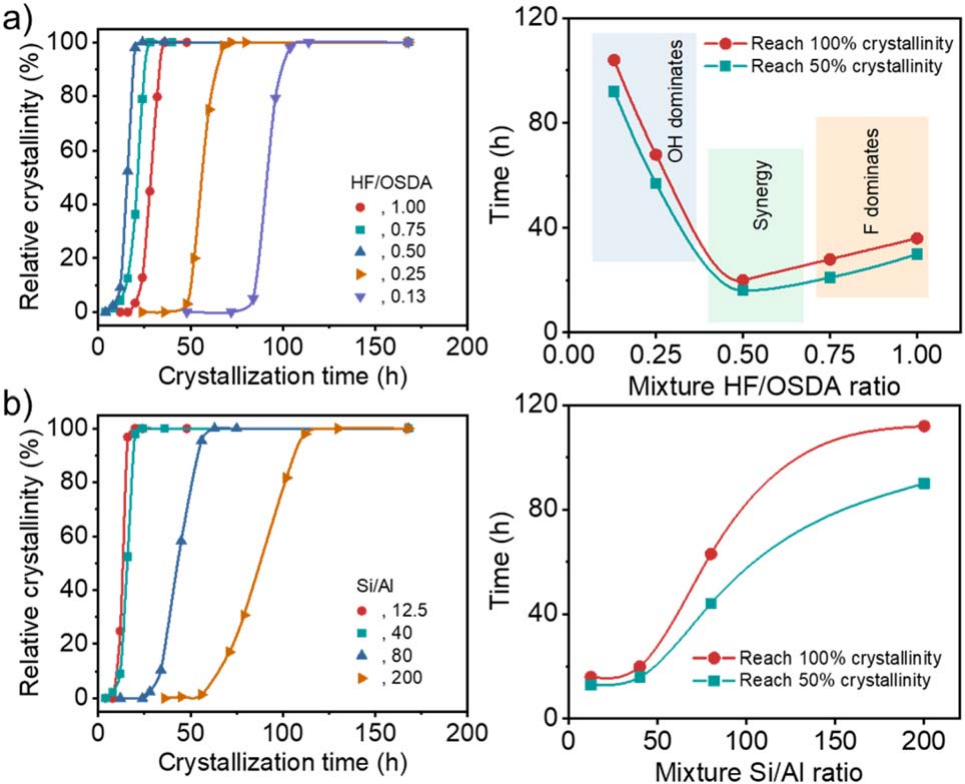
Crystallinity of a series of solid products isolated as a function of time (left) and synthesis time needed to reach 50 and 100% crystallinity as a function of mixture HF/OSDA (or mixture Si/Al) ratio (right), using starting mixtures (a) with the same Si/Al ratio (40) but different HF/OSDA ratios (0.13–1.00) and (b) with the same HF/OSDA ratio (0.50) but different Si/Al ratios (12.5–200).

Under all conditions described above, we were able to crystallize beta zeolite in pure form (Fig. S1). However, the kinetics study (Fig. 1a) showed that while at the standard HF/OSDA ratio of 1.00, fully crystalline beta was obtained in 48 h, HF/OSDA = 0.50 led to a faster synthesis time (20 h). Unexpectedly, a further ratio decrease to 0.13 resulted in a considerable increase in synthesis time (*ca.* 5 days). It thus appears that when none of the mineralizers prevails, there is a synergistic effect (Fig. 1a, right) that maximizes the beta zeolite nucleation and growth rates, while this effect is lost upon predominance of one mineralizer (F^−^ at close to neutral pH and OH^−^ at alkaline pH (HF/OSDA ≤ 0.25), respectively; see below). We also investigated the crystallization kinetics of synthesis mixtures with a fixed HF/OSDA optimum ratio (0.50) but different Si/Al ratios (12.5–∞). Interestingly, the larger the Al content in the synthesis mixtures, the shorter the crystallization time, which is opposed to the trend observed for the synthesis under standard fluoride conditions (Fig. 1b),^19^ again demonstrating the synergistic effect of the fluoride-deficient approach. We also note that not only does the “induction period” (the time before any powder X-ray diffraction (PXRD) crystallinity is observed) become longer, but also the crystallization rate slows as the Si/Al ratio increases. As a result, a pure-silica (Si/Al = ∞) synthesis mixture with HF/OSDA = 0.50 was found to remain amorphous even after heating at 140 °C for 2 weeks, although it yielded a mixture of beta and ZSM-5 after prolonged heating for 3 weeks. This suggests that the counterbalance of OSDA cations by single framework negative charges (*i.e.* [AlO_4/2_]^−^ tetrahedra) is more favorable than by connectivity defects.

**Fig. 2:**
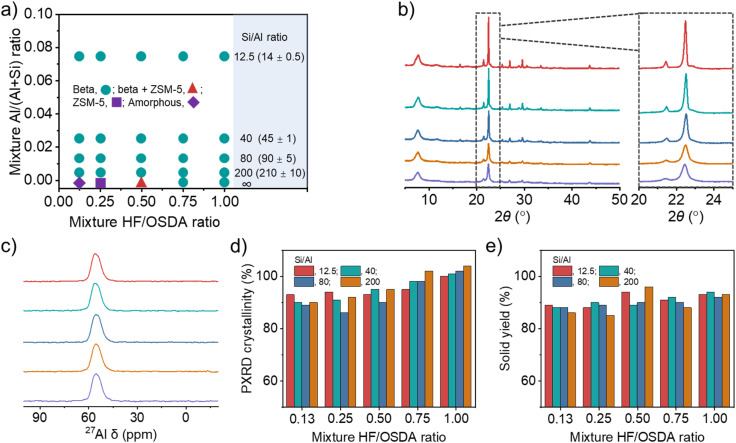
(a) Solid products obtained from composition 45.0TEAOH·*x*HF·*y*Al_2_O_3_·80.0SiO_2_·720H_2_O, where *x* and *y* are varied between 5.8 ≤ *x* ≤ 45.0 and 0 ≤ *y* ≤ 3.2, respectively, after 7 (cyan), 21 (red), or 28 (purple) days of crystallization. Values in parentheses are Si/Al ratios determined by elemental analysis. (b) PXRD patterns and (c) ^27^Al MAS NMR spectra of as-made beta zeolites obtained using mixtures with the same Si/Al ratio (40) but different HF/OSDA ratios (from bottom to top: 0.13, 0.25, 0.50, 0.75 and 1.00). (d) Crystallinity and (e) solid yield as a function of mixture HF/OSDA ratio at different Si/Al ratios (12.5–200).

Fig. 2a shows the phase products isolated at different time points (1–4 weeks) during zeolite crystallization using synthesis mixtures with different Si/Al (12.5–∞) and HF/OSDA (0.13–1.00) ratios. As previously reported,^24^ the Si/Al ratio of the starting mixture yielding pure beta at HF/OSDA = 1.00 was wide (12.5–∞). However, no pure-silica beta was obtained at HF/OSDA ≤ 0.50. More interestingly, pure-silica ZSM-5 (framework type MFI) was the phase that crystallized at HF/OSDA = 0.25 after heating at 140 °C for three weeks (Fig. S2 and S3). On the other hand, a further decrease in the HF/OSDA ratio to 0.13 at pure-silica composition gave no crystalline product even after 4 weeks. This implies that the phase selectivity of the crystallization in the presence of OSDAs can be altered not only by increasing HF content in the starting mixture,^16–20^ but also by decreasing this synthesis parameter. Raman spectroscopy shows that the TEA^+^ ions in pure-silica beta and ZSM-5 adopt *tg.tg* and *tt.tt* conformations, respectively (Fig. S2), where *t* and *g* refer to *trans* and *gauche* conformers, respectively, as shown by Schmidt *et al.*^36^ To date, there are two examples where pure ZSM-8,^37^ an MFI-type zeolite, has been synthesized using TEA^+^ as an OSDA: one in hydroxide media (basic conditions) and the other in fairly acidic (pH = 2.9–4.8) fluoride media.^38,39^ Our pure-silica ZSM-5 is different from these examples in that it crystallizes under basic conditions, although the synthesis has been performed in the presence of fluoride (Fig. S3).

Fig. 2b shows the PXRD patterns of beta zeolites crystallized at 140 °C for 7 days using starting mixtures with the same Si/Al ratio (40) but different HF/OSDA ratios (0.13–1.00). The pattern of the product synthesized at HF/OSDA = 1.00 shows sharp and broad reflections, typical of a highly crystalline beta.^6,12–24^ All X-ray peaks become broader and smaller with decreasing HF/OSDA ratio from 1.0 to 0.13, with high N_2_ BET surface areas (Table 1 and Fig. S4), but no appearance of new peaks from other crystalline phases, which was also observed for the other series of beta zeolites synthesized at the same Si/Al ratio (12.5, 80, or 200) but different HF/OSDA ratios (Fig. S5). The ^27^Al MAS NMR spectra of as-made beta zeolites, as well as of the corresponding but calcined ones, were characterized by only one resonance around 55 ppm due to tetrahedral framework Al atoms (Fig. 2c and S6–S7), and their measured crystallinities were always above 85% (Fig. 2d), suggesting that peak broadening is due to a decrease in crystal size. The yield of beta zeolites was also found to be high (≥85%), regardless of the mixture Si/Al and HF/OSDA ratios (Fig. 2e), in good agreement with the fact that the Si/Al ratio of each final solid product is quite similar to that of the starting mixture (Table 1 and Fig. 2a).

**Table 1:** Physical properties of two representative series of as-made beta zeolites

Sample ID	Hydrated unit cell composition	Si/Al ratio	Average crystal size (nm)	BET surface area (m^2^ g^−1^)	Micropore volume (cm^3^ g^−1^)	Mesopore volume (cm^3^ g^−1^)
Beta-40-0.13	|TEA_5.8_F_0.9_OH_3.5_|[Si_62.6_Al_1.4_O_128_]	44 (45)	40 (±3)	560 (180)	0.18	0.75
Beta-40-0.25	|TEA_6.1_F_1.3_OH_3.4_|[Si_62.6_Al_1.4_O_128_]	45 (44)	40 (±3)	570 (160)	0.18	0.59
Beta-40-0.50	|TEA_6.0_F_2.7_OH_1.9_|[Si_62.6_Al_1.4_O_128_]	46 (47)	75 (±5)	570 (140)	0.21	0.44
Beta-40-0.75	|TEA_5.9_F_3.3_OH_1.2_|[Si_62.6_Al_1.4_O_128_]	46 (48)	300 (±20)	570 (130)	0.20	0.31
Beta-40-1.00	|TEA_6.0_F_3.9_OH_0.7_|[Si_62.6_Al_1.4_O_128_]	44 (45)	1800 (±100)	560 (130)	0.20	0.28
Beta-12.5-0.50	|TEA_5.8_F_0.2_OH_1.5_|[Si_59.9_Al_4.1_O_128_]	14.5	50 (±5)	580 (200)	0.18	0.51
Beta-80-0.50	|TEA_6.2_F_4.5_OH_1.0_|[Si_63.3_Al_0.7_O_128_]	93	200 (±20)	570 (130)	0.20	0.32
Beta-200-0.50	|TEA_5.9_F_5.1_OH_0.5_|[Si_63.7_Al_0.3_O_128_]	216	1200 (±50)	570 (140)	0.20	0.20
Beta-∞-1.00	|TEA_6.0_F_6.0_|[Si_64_O_128_]	∞	2800 (±100)	550 (100)	0.20	0.13
beta(c)-12.5^f^		12.5	40 (±4)	560 (190)	0.16	0.49

a The last two numbers in the sample ID are the Si/Al and HF/OSDA ratios of the synthesis mixture used in zeolite crystallization, respectively.

b Determined from a combination of elemental and thermal analyses and ^19^F MAS NMR spectroscopy. The number of F^−^ions per unit cell of each aluminosilicate zeolite was determined by comparing the total area of ^19^F resonances with that of beta-∞-1.00 (pure-silica beta zeolite synthesized at HF/OSDA = 1.00) that was assumed to have six F^−^ ions per unit cell. OH^−^has been introduced to make as-made zeolites electrically neutral.

c Determined by elemental analysis. The values in parentheses are the Si/Al ratio after ammonium ion exchange.

d Determined by FE-SEM/TEM. The values in parentheses are the standard deviations of the crystal size.

e Calculated from N_2_ adsorption data of the proton form of each beta zeolite. The values in parentheses are the external surface areas. Micropore and mesopore volumes were calculated by *t*-Plot and BJH methods, respectively.

f A commercial beta zeolite with Si/Al = 12.5 (CP814E) obtained from Zeolyst.

**Fig. 3:**
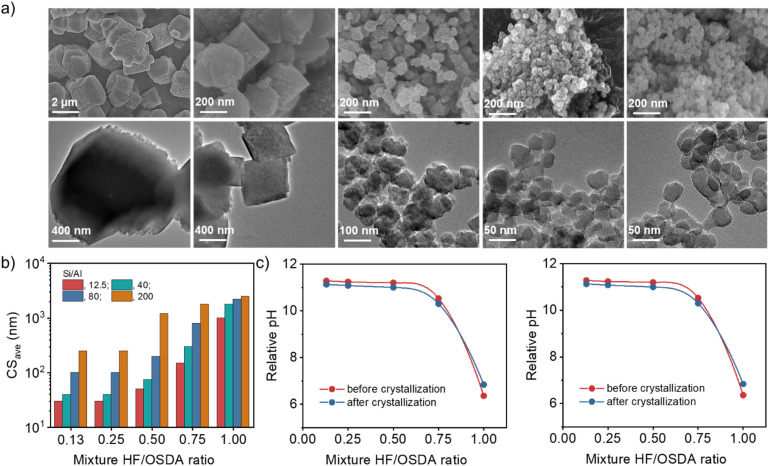
(a) FE-SEM (top) and TEM (bottom) images of as-made beta zeolites obtained using mixtures with the same Si/Al ratio (40) but different HF/OSDA ratios (from left to right: 1.00, 0.75, 0.50, 0.25 and 0.13) after heating at 140 °C for 7 days. (b) Average crystal sizes (CS_ave_) of zeolites obtained using mixtures with different Si/Al ratios (12.5–200) and HF/OSDA ratios (0.13–1.00). (c) Relative pH value as a function of HF/OSDA ratio of starting mixtures with Si/Al ratios of 12.5 (left) and 40 (right) before and after crystallization.

Fig. 3a shows the FE-SEM and TEM images of a series of beta zeolites synthesized at the same Si/Al ratio (40) but different HF/OSDA ratios (0.13–1.00), where no amorphous phase is evident. As the HF/OSDA ratio in the starting mixture decreases from 1.00 to 0.25, the crystals become significantly smaller (from *ca.* 1800 to 40 nm), less faceted and thinner, but are still discrete. No additional decrease in average crystal size was caused by further decreasing the HF/OSDA ratio to 0.13 (Fig. 3b). The observation holds at other Si/Al ratios (Fig. S8). For example, the mixtures with Si/Al = 12.5 and 80 gave the smallest beta crystals (30 and 100 nm, respectively) at HF/OSDA = 0.25, although considerably bigger ones (*ca.* 250 nm) were obtained at Si/Al = 200 and HF/OSDA = 0.25 (Fig. 3b). Decreasing the HF/OSDA ratio to 0.13 led to no further decrease in size, at any given Si/Al ratio. It thus appears that while there is a critical level of F^−^ concentration (HF/OSDA ≤ 0.25) in the starting mixture leading to the smallest crystal size, the average size of the smallest beta crystals increases with increasing the mixture Si/Al ratio.

Fig. 3c shows the relative pH value as a function of HF/OSDA ratio, before and after beta crystallization, of starting mixtures with Si/Al ratios of 12.5 and 40. For both ratios, the mixtures are slightly acidic at HF/OSDA = 1.00 (*i.e.* under standard fluoride conditions) and have become slightly less acidic after crystallization. However, a decrease in HF/OSDA ratio to 0.75 has already led them to be basic because the TEAOH amount (OSDA/SiO_2_ = 0.56) in all synthesis mixtures was kept constant. Their relative pH value reaches the maximum at HF/OSDA = 0.50 and remains almost unchanged at ratios up to 0.13, which is essentially the same trend observed not only for the starting mixtures with higher Si/Al ratios (80 or 200) (Fig. S9), but also for the decrease in beta crystal size (Fig. 3b) and for the decrease in crystallization time. The synergistic effect is thus observed for HF/OSDA values that maximize the OH^−^ concentration without compromising too much the F^−^ concentration. For a given Si/Al ratio, the pH determines the crystal size, which can be rationalized by the higher nucleation rate observed at higher pH.^40^ The almost constant pH and crystal size for HF/OSDA ≤ 0.50 are likely due to a buffering effect of silica.^41^ However, accurate elucidation of the effects of mixture alkalinity on the nucleation and crystal growth of beta zeolite is not an easy task, mainly because the effect of fluoride itself cannot be ruled out. Moreover, since the synthesis of the pure-silica analog is possible only in a high range (0.75–1.00) of HF/OSDA ratios (Fig. 2a), there appears to be a minimum level (Si/Al ≤ 200) of Al in order to accelerate beta crystallization at the optimum HF/OSDA ratios (≤0.50; *i.e.* under more basic conditions).

### 3.2 Defect characterization

**Fig. 4:**
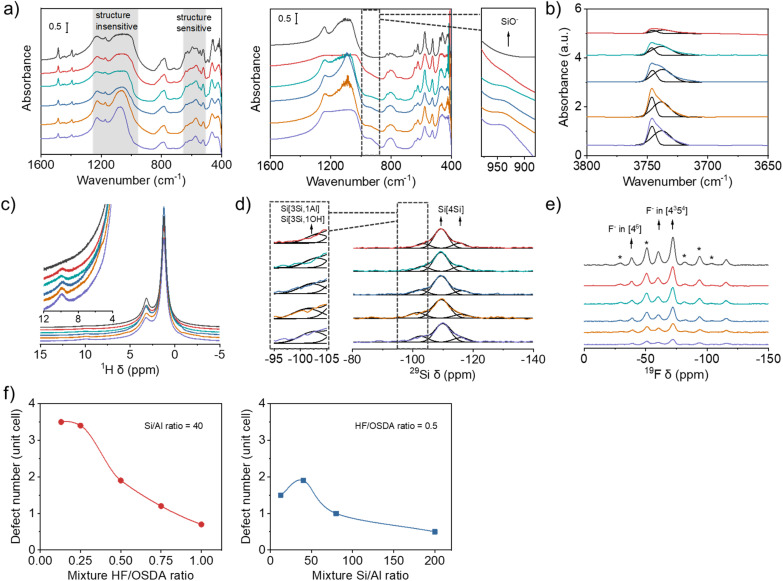
(a) IR spectra in the framework vibration region of the as-made (left) and calcined (right) beta zeolites obtained using synthesis mixtures with the same Si/Al ratio (40) but different HF/OSDA ratios (from bottom to top: 0.13, 0.25, 0.50, 0.75 and 1.00) and (b) IR spectra in the OH stretching region of the proton form of the above series of zeolites. (c) ^1^H, (d) ^29^Si and (e) ^19^F MAS NMR spectra of the same as-made zeolites. Spinning sidebands are indicated by asterisks. In (a), (c) and (e), the spectra of pure-silica beta zeolite synthesized under standard fluoride conditions (HF/OSDA = 1.00) are given as a top trace in each panel. (f) Number of SiO^−^⋯HOSi defects per unit cell as a function of mixture HF/OSDA ratio at a fixed Si/Al ratio (40) (left) and of mixture Si/Al ratio at a fixed HF/OSDA ratio (0.50) (right).

Fig. 4a shows the IR spectra in the framework vibration region of the as-made and calcined forms of beta zeolites obtained using synthesis mixtures with the same Si/Al ratio (40) but different HF/OSDA ratios. Although there are several overlapping bands in the region 500–650 cm^−1^, the overall bands in this region are highly characteristic of zeolite beta, which becomes clearer for calcined beta zeolites.^22–24^ Their similar relative intensities suggest that all the beta zeolites are equally well-crystallized, regardless of differences in crystal size (Fig. 3b) or measured crystallinity (Fig. 2c). This is also the case for a series of beta zeolites obtained using synthesis mixtures with the same HF/OSDA ratio (0.50) but different Si/Al ratios (12.5–200) (Fig. S10). Of particular interest is the appearance of a shoulder around 940 cm^−1^ in the IR spectra of calcined (thus the proton form) aluminosilicate beta zeolites synthesized at the same mixture Si/Al ratio (40) but different HF/OSDA ratios, assignable to the stretching vibration of SiO^−^ defects,^42^ which is not observed for calcined pure-silica beta synthesized at HF/OSDA = 1.00. This band gradually increases as the mixture HF/OSDA ratio decreases to 0.13 (Fig. 4a). Fig. 4b shows the IR spectra in the OH region of a series of H-beta-40-*n* zeolites (*n* = HF/OSDA = 0.13–1.00). The bands at 3745 and 3735 cm^−1^ are assigned to terminal and internal SiOH groups, respectively.^43^ An increase in intensity of the latter band with decreasing *n* to 0.25 indicates an increase in internal SiOH concentration. However, there are no noticeable differences in the intensity of the band at 3610 cm^−1^ due to BAS-OH groups (Fig. S11), in good agreement with the similarity in their Si/Al ratios (Table 1).

To gain further evidence for the presence of internal SiO^−^⋯HOSi defects in aluminosilicate beta zeolites synthesized here, we collected the ^1^H MAS NMR spectra of their as-made form (Fig. 4c). While the ^1^H resonances at 1.2 and 3.2 ppm are mainly due to the protons of the methyl and methylene groups of TEA^+^ cations, respectively, the very weak resonance at 9.9 ppm can be attributed to SiOH groups involved in relatively strong hydrogen bonds with neighboring SiO^−^ or SiOH groups, thus evidencing the existence of internal SiOH groups.^44^ While this resonance is again missing in the spectrum of as-made pure-silica beta synthesized at HF/OSDA = 1.00, it becomes slightly stronger as *n* decreases to 0.13, similar to the IR band around 940 cm^−1^ of the corresponding calcined zeolites (Fig. 4a). This ^1^H chemical shift corresponds to an O⋯O distance of 2.7 Å,^45^ consistent with internal SiOH groups. Groups with weaker hydrogen bonding can resonate at higher fields and appear overlapped with the signals of the OSDA cation. A combination of IR and ^1^H MAS NMR spectroscopy also shows the presence of internal SiO^−^⋯HOSi defects in as-made aluminosilicate beta zeolites synthesized at a fixed HF/OSDA ratio (0.50) but different Si/Al ratios (12.5–200) (Fig. S10 and S12).

The presence of internal defects in as-made beta-40-*n* zeolites is also supported by ^29^Si MAS NMR spectroscopy. Although there are no notable differences in the overall ^29^Si line shape, curve deconvolution indicates that the relative intensity of the resonance appearing around −103 ppm, assignable to Si(3Si, 1Al) and Si(3Si, 1OH) species,^23^ gradually increases as *n* decreases to 0.13 (Fig. 4d and Table S1). This is attributed to an increase in SiO^−^⋯HOSi defect concentration, because these zeolites have essentially the same Si/Al ratio (45 ± 1; Table 1). Similar results were observed on the corresponding ^1^H–^29^Si CP MAS NMR spectra (Fig. S13). The ^19^F MAS NMR spectra of as-made beta-40-*n* zeolites are compared with the spectrum of as-made pure-silica beta synthesized at HF/OSDA = 1.00 in Fig. 4e. All the spectra are essentially identical, except for a decreased overall intensity as *n* (HF/OSDA ratio) decreases. The ^19^F resonance around −40 ppm, assigned to F^−^ occluded in double 4-ring (*d*4*r*; [4^6^]) cages,^9,10^ demonstrates the existence of polymorphs other than A and B in these zeolites (C, D or E) due to the lack of *d*4*r*s in the former two polymorphs.^6^ For the sake of comparison and simplicity, here we refer to a 64 T-atom unit cell.

Si–OH and SiO^−^ groups may arise from two origins: terminal groups (increasing as the crystal size decreases) and internal groups that may be necessary for charge balance when the concentration of fluoride decreases and that usually appear in about a fourfold excess.^36^ It has been repeatedly shown that, for materials prepared by the fluoride route, the beta unit cell retains six TEA^+^ ions, regardless of the framework Si/Al ratio,^33,46,47^ and thus a maximum of six F^−^ anions at pure-silica composition. As Al is incorporated into the beta framework, the number of F^−^ ions should decrease because of the counterbalance of OSDA cations by [AlO_4/2_]^−^ tetrahedra. Table 1 compares the unit cell compositions of as-made beta-40-*n* and beta-*m*-0.50, where *m* = Si/Al = 12.5–200, which were determined from a combination of elemental and thermal analyses and ^19^F MAS NMR spectroscopy (Fig. 4e and S14–S15). The former series of zeolites have essentially the same number (Si/Al = 45 ± 1) of Al atoms and TEA^+^ cations, around 1.4 and 6.0, respectively, per unit cell, while the F^−^ concentration decreases from 3.9 to 0.9 per unit cell with decreasing *n* from 1.00 to 0.13, leading to more and more OH^−^ ions, actually representing SiO^−^ defects, introduced in Table 1 for charge balance. The variation of Al atoms, TEA^+^ cations and F^−^ concentration in the latter series of zeolites also caused the difference in the concentration of SiO^−^ in each unit cell. To better understand the change, we plotted SiO^−^ number per unit cell of the beta zeolites with respect to the HF/OSDA and Si/Al ratios in the synthesis mixture (Fig. 4f). We note that the SiO^−^ defect concentration significantly increases in the beta zeolites as the HF/OSDA ratio decreases and the maximum concentration can reach *ca.* 3.5 per unit cell when the HF/OSDA ratio decreases to 0.25. In addition, at a fixed HF/OSDA ratio of 0.50, the SiO^−^ defect number varies with the Si/Al ratio, and the maximum value appears in the beta zeolite obtained using a synthesis mixture with a Si/Al ratio of 40.

### 3.3 Wet CH_4_ combustion activity

We prepared a series of 3.0Pd/H-beta-12.5-*n*, 3.0Pd/H-beta-40-*n* and 1.5Pd/H-beta-80-*n* catalysts with different Si/Al ratios and support crystal sizes (Table S2), where *n* = HF/OSDA = 0.25–1.00, and compared their catalytic performance in order to investigate the effects of support crystal size and defect concentration on the CH_4_ combustion activity of supported Pd catalysts. Here beta zeolites synthesized at HF/OSDA = 0.13 were not used as Pd supports because their average crystal sizes, as well as defect concentrations, are essentially the same as those of zeolites synthesized at HF/OSDA = 0.25, regardless of the mixture Si/Al ratio (Fig. 3b and 4f). Also, zeolites synthesized at Si/Al = 200 were not included due to their low Pd^2+^ exchange capacities (<1.0 wt% Pd).

**Fig. 5:**
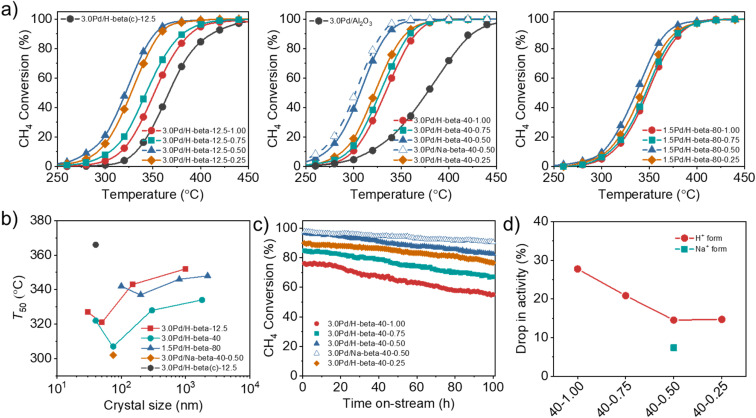
(a) CH_4_ conversion as a function of reaction temperature over a series of 3.0Pd/H-beta-12.5-*n* (left), 3.0Pd/H-beta-40-*n* (middle) and 1.5Pd/H-beta-80-*n* (right) catalysts with different average support crystal sizes, where *n* is the HF/OSDA synthesis ratio. Also given are the data of 3.0Pd/Na-beta-40-0.50, 3.0Pd/H-beta(c)-12.5 and 3.0Pd/Al_2_O_3_. The latter catalyst was prepared using commercial beta zeolite (Si/Al = 12.5) as a support. (b) *T*_50_ as a function of the average support crystal size of the above series of Pd beta catalysts. (c) CH_4_ conversion at 350 °C as a function of time on stream over 3.0Pd/H-beta-40-*n* (*n* = 0.25–1.00) and 3.0Pd/Na-beta-40-0.50 catalysts. Reactant feed composition: 1500 ppm CH_4_, 5% O_2_ and 10% H_2_O balanced with N_2_ at a GHSV of 100 000 h^−1^. (d) Percentage drops in activity of 3.0Pd/H-beta-40-*n* and 3.0Pd/Na-beta-40-0.50 catalysts after 100 h on stream at 350 °C.

The catalytic data in Fig. 5a reveal that the *T*_50_ value of supported Pd catalysts is always higher in the support order Pd/H-beta-0.50 < Pd/H-beta-0.25 < Pd/H-beta-0.75 < Pd/H-beta-1.00, where the last number is the HF/OSDA ratio of starting mixtures, regardless of the support Si/Al ratio and Pd loading level. Therefore, beta zeolite with the second smallest average crystal size (50, 75, or 100 nm; Fig. 3b) in each series of zeolites with similar Si/Al ratios (*i.e.* 14, 45, or 90) was found to be the best support for Pd. In addition, when the Pd loading level was fixed to be 3.0 or 1.5 wt%, a considerably lower *T*_50_ value was observed for beta zeolites with Si/Al = 45, in line with our recent work.^33^ As a result, the 3.0Pd/H-beta-40-0.50 catalyst gave the lowest *T*_50_ value (305 °C) among the proton form of all catalysts studied here, which was much lower than the value (380 °C) of a reference 3.0Pd/Al_2_O_3_ catalyst (Fig. 5a). Also, its *T*_50_ value decreased slightly when Na^+^ was post-exchanged (Fig. 5b). It is interesting to note here that the *T*_50_ value (320 °C) of 3.0Pd/H-beta-12.5-0.50 is considerably lower than that (365 °C) of 3.0Pd/H-beta(c)-12.5, *i.e.* a 3.0 wt% Pd catalyst supported on the commercial beta zeolite with a similar Si/Al ratio (12.5 *vs.* 14) and nanocrystal size (*ca.* 40 nm) (Fig. S16). The origin of this will be discussed later.

We also investigated the long-term performance of a series of 3.0Pd/H-beta-40-*n* catalysts with essentially the same support Si/Al ratios (45 ± 1) but different average support crystal sizes (40–1800 nm) at 350 °C (Fig. 5c). All catalysts showed no rapid decrease in CH_4_ conversion due to site blocking during 100 h on stream in the presence of 10% H_2_O. However, because of the hydroxylation of active PdO to less active Pd(OH)_2_ by H_2_O adsorption at temperatures below 450 °C,^31,48^ there is a gradual decrease in conversion (Fig. 5c), which was also observed for 3.0Pd/H-beta(c)-12.5 and 3.0Pd/H-beta-12.5-0.75 (Fig. S17). Interestingly, the percentage drop in activity was smallest for 3.0Pd/H-beta-40-0.50 with the second smallest average crystal size (75 nm) in this series of Pd/beta catalysts (Fig. 5d). No further decrease in activity drop was observed for 3.0Pd/H-beta-40-0.25 with the smallest average crystal size (40 nm), whereas the CH_4_ conversion over the latter catalyst is always lower by *ca.* 7% than that over the former one during the period of 100 h on stream. These results clearly show the existence of an optimal zeolite crystal size that maximizes the long-term stability of supported Pd catalysts for wet CH_4_ combustion, similar to their activity case (Fig. 5b). It should be noted that the higher defect concentration the zeolite support (Table 1), the higher long-term stability the supported Pd catalyst. In fact, SiOH nests in zeolites have been recently suggested to protect the PdO nanoparticles from sintering during the reaction or slow down the sintering process.^49^ We also found that post-exchange of Na^+^ cations in 3.0Pd/H-beta-40-0.50 followed by air calcination at 500 °C led to a further decrease in activity drop and thus an enhancement of catalyst stability due to the considerable increase in Pd surface area.^33^

### 3.4 Catalyst characterization

**Fig. 6:**
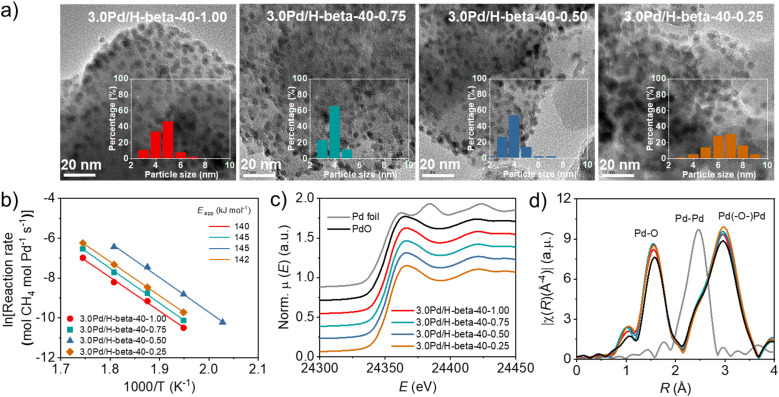
(a) TEM images, (b) Arrhenius plots and apparent activation energies for wet CH_4_ combustion, (c) Pd *K*-edge XANES spectra and (d) *k*^3^-weighted EXAFS Fourier transforms of a series of 3.0Pd/H-beta-40-*n* catalysts with different average support crystal sizes (for *n*, see Fig. 5a). Also given are Pd foil and PdO references in (c and d).

The PXRD patterns of the above series of Pd/beta catalysts with essentially the same support Si/Al ratios (14 ± 0.5, 45 ± 1, or 90 ± 5) but different average support crystal sizes (Fig. 3b) show no noticeable differences in the broad feature of an X-ray peak around 2*θ* = 33.8° corresponding to the (101) reflection of PdO, despite significant differences in the average support crystal size (Fig. S18). However, the TEM images in Fig. 6a show that while the PdO particles in 3.0Pd/H-beta-40-1.00, 3.0Pd/H-beta-40-0.75 and 3.0Pd/H-beta-40-0.50 have average diameters (4–5 nm) quite similar to one another, those in 3.0Pd/H-beta-40-0.25 are bigger in diameter (*ca.* 7 nm) and broader in size distribution. This explains why the *T*_50_ value (320 °C) of 3.0Pd/H-beta-40-0.25 is higher than that (305 °C) of 3.0Pd/H-beta-40-0.50 (Fig. 5b), despite its smaller average support crystal size (40 *vs.* 75 nm). A similar interpretation can be given for the poor catalytic performance of 3.0Pd/H-beta(c)-12.5 with a smaller average crystal size of 40 nm (Fig. S16) and a lower defect concentration (Fig. S10 and S19). Therefore, we speculate that if the zeolite support is too small (≤50 nm), the size of supported PdO particles can be larger, probably due to the relatively faster heat transfer from the zeolite support to the ion-exchanged Pd precursor species during the air calcination step for PdO formation.

**Fig. 7:**
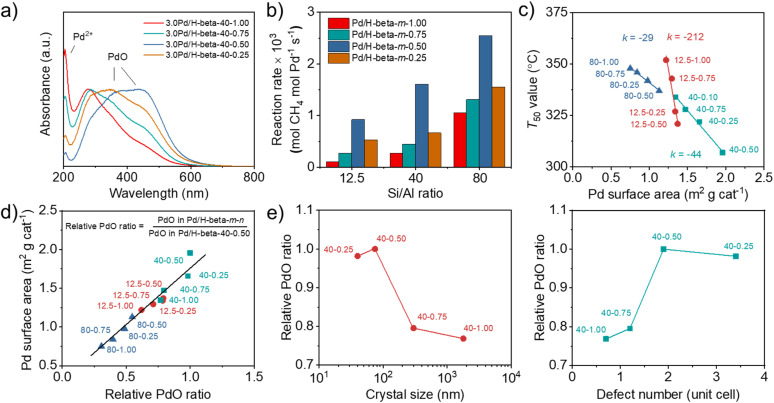
(a) UV-vis DR spectra of 3.0Pd/H-beta-40-*n* catalysts. (b) Reaction rates at 280 °C over 3.0Pd/H-beta-12.5-*n*, 3.0Pd/H-beta-40-*n* and 1.5Pd/H-beta-80-*n* catalysts. (c) *T*_50_ as a function of Pd surface area and (d) Pd surface area as a function of relative PdO ratio of 3.0Pd/H-beta-12.5-*n*, 3.0Pd/H-beta-40-*n* and 1.5Pd/H-beta-80-*n* catalysts. (e) Relative PdO ratio of 3.0Pd/H-beta-40-*n vs.* zeolite crystal size (left) and zeolite defect number per unit cell (right).

When calculated based on the slope of Arrhenius plots for wet CH_4_ combustion over the series of 3.0Pd/H-beta-40-*n* catalysts, on the other hand, there were no significant differences (140–145 kJ mol^−1^) in the apparent activation energy (Fig. 6b). The same result was also observed for 3.0Pd/H-beta-12.5-*n* and 3.0Pd/H-beta-80-*n* catalysts (Fig. S20). In fact, Pd *K*-edge X-ray absorption near-edge structure (XANES) and extended X-ray absorption fine structure (EXAFS) spectroscopy show that 3.0Pd/H-beta-40-*n* catalysts with *n* = 0.25–1.00 have similar coordination numbers and Pd (–O–)Pd distances (Fig. 6c, d, S21–S22 and Table S3). Also, all of their NH_3_ TPD profiles are characterized by a prominent peak around 270 °C due to NH_3_ desorption from intrazeolitic PdO species (Fig. S23). Therefore, it is clear that the observed differences in the combustion activity and stability (Fig. 5) can be attributed to those in the number of accessible PdO sites. This can be further supported by the UV-vis DR spectra in Fig. 7a and S24. All the spectra exhibit a sharp Pd^2+^ ← O charge-transfer (CT) band around 205 nm and three broad CT bands at 300–500 nm, assignable to the isolated Pd^2+^ ions in extra-framework charge-balancing positions and the small PdO clusters, respectively.^33,50^ It can be seen that the relative ratio of PdO/Pd^2+^ bands increases in the order of 3.0Pd/H-beta-40-1.00 < 3.0Pd/H-beta-40-0.75 < 3.0Pd/H-beta-40-0.25 < 3.0Pd/H-beta-40-0.50, which is exactly the same order as their *T*_50_ values and reaction rates (Fig. 5b and 7b). This again confirms the importance of zeolite crystal size. However, we speculate that the internal defects in H-beta-40-0.50 may also play a beneficial role in the catalytic performance of the supported Pd catalyst. The ion exchange capacity of high-silica zeolites has long been recognized to differ according to the concentration of their defects.^51^ Apparently, the electrostatic interactions of Pd^2+^ ions with [AlO_4/2_]^−^ tetrahedra should be considerably stronger than those with SiO^−^ groups that are part of the zeolite framework, because of the shorter cation–anion distance. If such is the case, the cations counterbalanced by internal zeolite defects would then be more easily oxidized to form PdO compared to those by [AlO_4/2_]^−^ tetrahedra.

We also compared the Pd surface areas of Pd/beta catalysts, determined using pulse CO chemisorption, with their respective *T*_50_ values (Fig. 7c). When the support Si/Al ratio is similar, the *T*_50_ value decreases linearly with increasing Pd surface area. More interestingly, the rate of *T*_50_ decrease was found to be smaller at a higher support Si/Al ratio. Since each series of Pd catalysts under comparison has similar support Si/Al ratios and Pd loadings but different average support crystal sizes (Fig. 2a, 3b and Table S2), the effects of support crystal size on the *T*_50_ value appear to be less significant at a higher support Si/Al ratio. We also determined the relative PdO ratios of all Pd/H-beta-40-*n* catalysts from O_2_ TPD (Fig. S25) by dividing the peak area of each catalyst by that of 3.0Pd/H-beta-40-0.50 and plotted them against their Pd surface areas (Fig. 7d). A linear relationship between these two quantities again shows that PdO is a major low-temperature (< 400 °C) active center for wet CH_4_ combustion.

Finally, the plots of relative PdO ratio *versus* average zeolite crystal size and defect concentration suggest the presence of an optimal defect concentration in zeolite supports (Fig. 7e), maximizing the relative PdO ratio of supported Pd catalysts, because the 3.0Pd/H-beta-40-0.50 catalyst with the second smallest crystal size and the medium defect concentration among the H-beta-40-*n* series (Table 1) has the largest relative PdO ratio among all Pd catalysts studied here (Fig. 7d). Clearly, decreasing the crystal size (or shortening the diffusion path) of beta zeolites with similar Si/Al ratios should be beneficial to Pd^2+^ ion exchange, because Pd^2+^ ions can access the ion exchange sites faster, especially the internal SiOH groups whose concentration is higher in smaller crystals. However, if the crystal size is too small (*i.e.* 3.0Pd/H-beta-40-0.25 with an average crystal size of *ca.* 40 nm; Table 1), this would then result in more severe PdO sintering during calcination at 500 °C (Fig. 6a), thus increasing *T*_50_ (Fig. 5b). On the other hand, the high defect concentration can facilitate the formation of PdO with a high PdO/Pd^2+^ ratio during calcination (Fig. 7a), probably due to the weaker electrostatic interactions of Pd^2+^ ions with SiO^−^ groups than with [AlO_4/2_]^−^ tetrahedra, giving a lower *T*_50_ value. Therefore, we speculate that the internal SiOH groups may play a beneficial role in the mitigation of intrazeolitic PdO sintering during wet CH_4_ combustion, thus contributing to the long-term catalyst stability, unlike the terminal SiOH groups, which can stabilize the PdO particles located only on the external surface of zeolite crystals by hydrogen bonding.

## 4. Conclusions

We have successfully established a simple but effective approach for synthesizing discrete beta zeolite nanocrystals with Si/Al ratios of 14–90 in the range 30–100 nm by decreasing the HF/OSDA ratio of starting synthesis mixtures from unity to 0.13. We found that for a given Si/Al ratio in the synthesis mixture, the crystal size decreases as the HF/OSDA ratio decreases from 1.00 to 0.25, while a further decrease to 0.13 leaves the crystal size unaffected. This parallels the variation in pH of the crystallizing medium and suggests that alkalinity is the main driving force that accelerates nucleation and controls the crystal size. At an alkaline HF/OSDA ratio of 0.50, the fluoride and hydroxide anions apparently act synergistically, leading to an acceleration of crystallization compared to higher and lower ratios. The use of a significantly smaller amount of toxic fluoride in the fast synthesis of aluminosilicate beta nanozeolites can alleviate the disadvantage of the fluoride route.

The overall characterization results demonstrate that charge balance of six TEA^+^ ions per unit cell of 64 T-atoms requires, in all cases tested (0.3 to 4.7 Al atoms per unit cell), the presence of connectivity defects when the mixture HF/OSDA ratio is lower than unity. After loading Pd on these beta zeolites obtained at different Si/Al (12.5–80) and HF/OSDA (0.13–1.00) ratios, the resulting catalysts showed significantly different performance in wet CH_4_ combustion. The 3.0Pd/Na-beta-40-0.50 catalyst with the second smallest crystal size (*ca.* 75 nm) and the medium defect concentration (*ca.* 2 per unit cell) among the 3.0Pd/H-beta-40-*n* series with a framework Si/Al ratio of 45 was characterized by a low light-off temperature of 300 °C and a long catalyst stability of 100 h with >90% CH_4_ conversion at 350 °C), mainly due to its highest relative PdO ratio. This is one of the best results ever obtained using zeolite-supported Pd catalysts. The concept of the fluoride-deficient approach presented here is applicable to the synthesis of other industrially important high-silica zeolites on the nanometer scale, including MFI, CHA and LTA nanozeolites, the details of which will be given elsewhere.

## Author contributions

X. T.: methodology, investigation, writing – original draft. M. A. C.: conceptualization, writing – review & editing, funding acquisition. S. B. H.: conceptualization, supervision, writing – review & editing, project administration, funding acquisition.

## Conflicts of interest

There are no conflicts to declare.

## Supplementary Material

SC-OLF-D5SC04097C-s001

## Data Availability

The data supporting this article have been included as part of the supplementary information (SI). Supplementary information is available. See DOI: https://doi.org/10.1039/d5sc04097c.
