# Supplementary material for: Synergistic zeolite synthesis via a fluoride-deficient mixed approach
Source: Chem Sci. 2025 Oct 13;16(44):21028–40. doi: 10.1039/d5sc04097c (PMC12516862; doi:10.1039/d5sc04097c)
Supplement: SC-016-D5SC04097C-s001 [file SC-016-D5SC04097C-s001.pdf]

Supplementary Information (SI) for Chemical Science.
This journal is © The Royal Society of Chemistry 2025

Supplementary Information

Synergistic zeolite synthesis via a fluoride-deficient mixed approach

Xuechao Tan, [a] Miguel A. Camblor [b] and Suk Bong Hong *[a]

[a] *Center for Ordered Nanoporous Materials Synthesis, Division of Environmental Science and Engineering, POSTECH, Pohang 37673, Korea*

[b] *Instituto de Ciencia de Materiales de Madrid (ICMM), CSIC, Madrid 28049, Spain*

*Corresponding author. *E-mail*: sbhong@postech.ac.kr

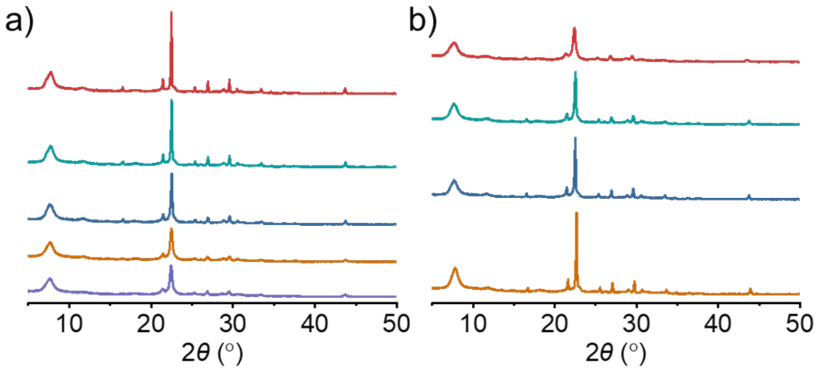

Fig. S1 PXRD patterns of a series of solid products obtained from zeolite syntheses under rotation (60 rpm) at 140 °C, using starting mixtures (a) with the same Si/Al ratio (40) but different HF/OSDA ratios (from bottom to top; 0.13, 0.25, 0.50, 0.75 and 1.00) and (b) with the same HF/OSDA ratio (0.50) but different Si/Al ratios (from top to bottom; 12.5, 40, 80 and 200). The synthesis time applied for each product is the same as that in Fig. 1.

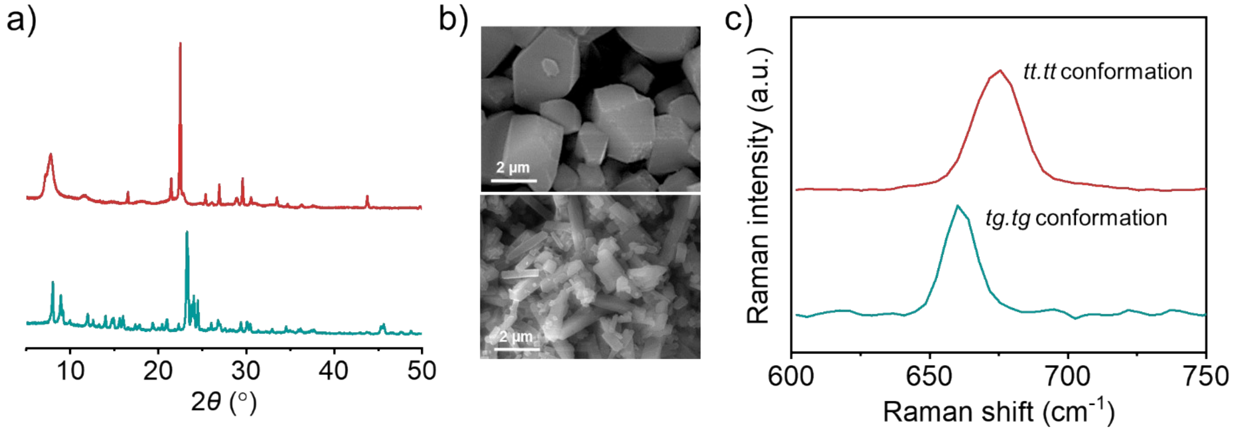

Fig. S2 (a) PXRD patterns, (b) SEM images and (c) Raman spectra of pure-silica beta and ZSM-5 zeolites synthesized at mixture HF/OSDA ratios of 1.00 and 0.25, respectively, using TEAOH as an OSDA. The synthesis time at 140 °C is 7 days for the former zeolite and 28 days for the latter one.

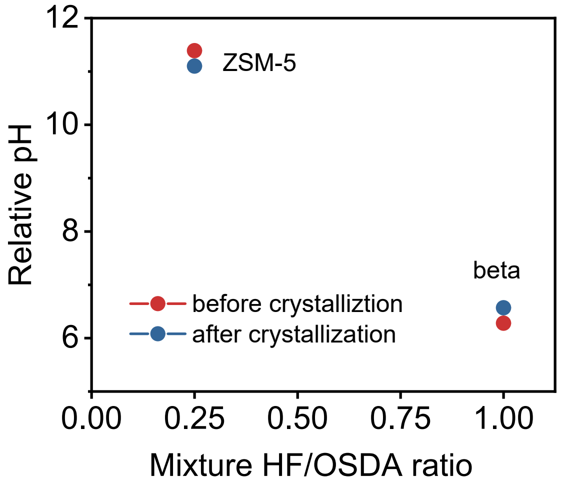

Fig. S3 Relative pH values of two pure-silica synthesis mixtures with HF/OSDA ratios of 1.00 and 0.50 and the corresponding final products (i.e. pure-silica beta and ZSM-5), respectively. Prior to the pH measurement, 1 g of each of the starting mixtures and final products was slurried with 10 mL of deionized water at room temperature for 0.5 h.

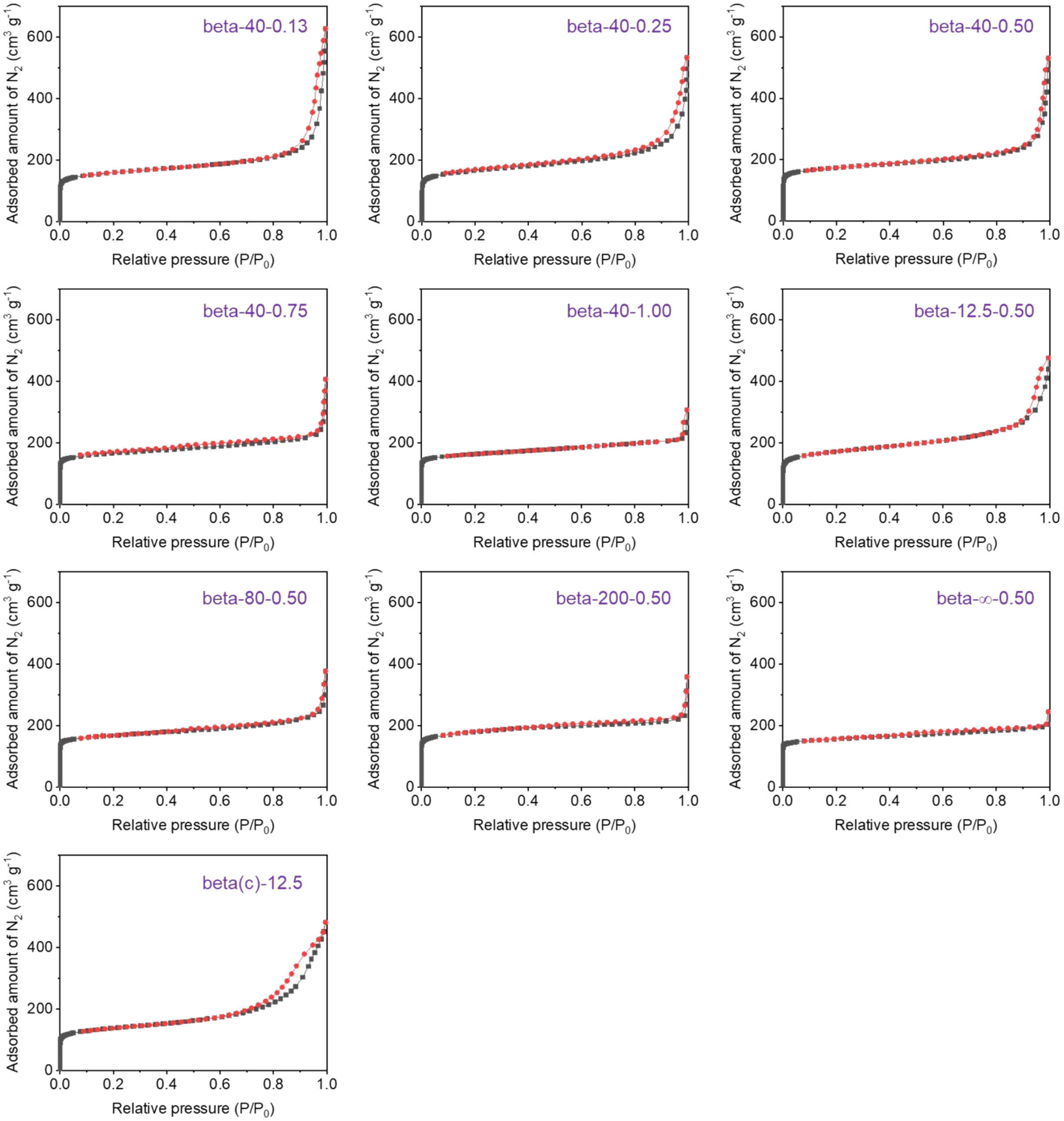

Fig. S4 N_2 adsorption isotherm curves of H-form beta zeolites obtained using mixtures with different Si/Al ratios (12.5 – ∞) and HF/OSDA ratios (0.23 – 1.00). Also given is the data of H-form commercial beta zeolite (Si/Al = 12.5).

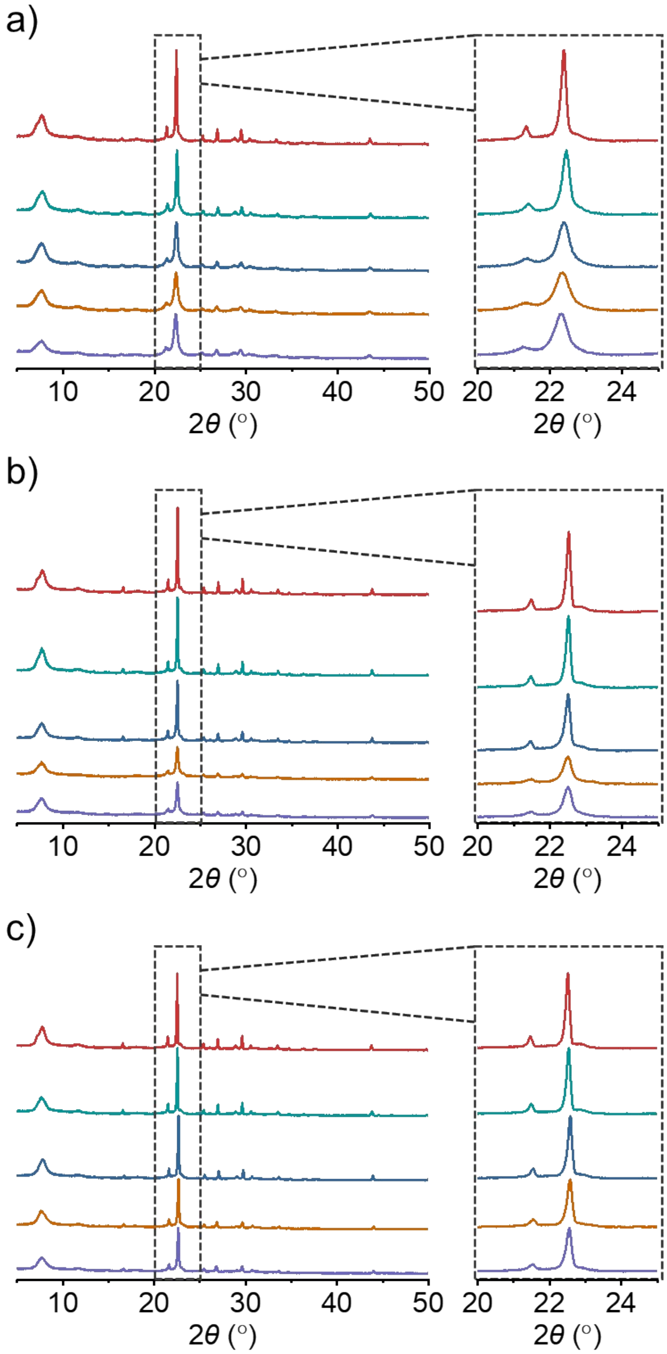

Fig. S5 PXRD patterns of beta zeolites synthesized at the same mixture Si/Al ratio (12.5, top; 80, middle; 200, bottom) but different mixture HF/OSDA ratios (from bottom to top: 0.13, 0.25, 0.50, 0.75 and 1.00) using TEAOH as an OSDA.

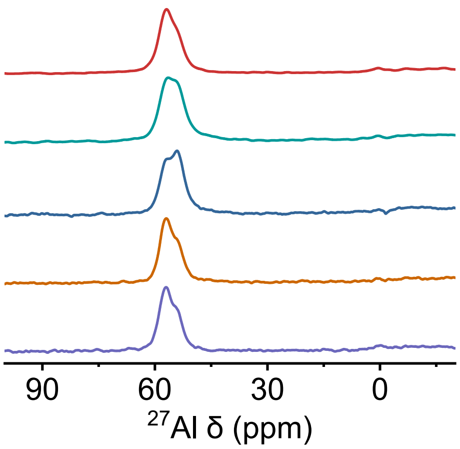

Fig. S6 ^{27}Al MAS NMR spectra of H-form beta zeolites obtained using mixtures with the same Si/Al ratio (40) but different HF/OSDA ratios (from bottom to top: 0.13, 0.25, 0.50, 0.75 and 1.00).

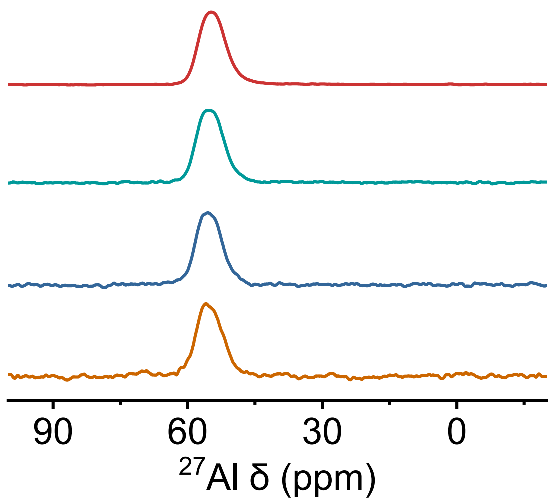

Fig. S7 ^{27}Al MAS NMR spectra of beta zeolites synthesized at the same mixture HF/TEAOH ratio (0.50) but different Si/Al ratios (from top to bottom: 12.5, 40, 80 and 200).

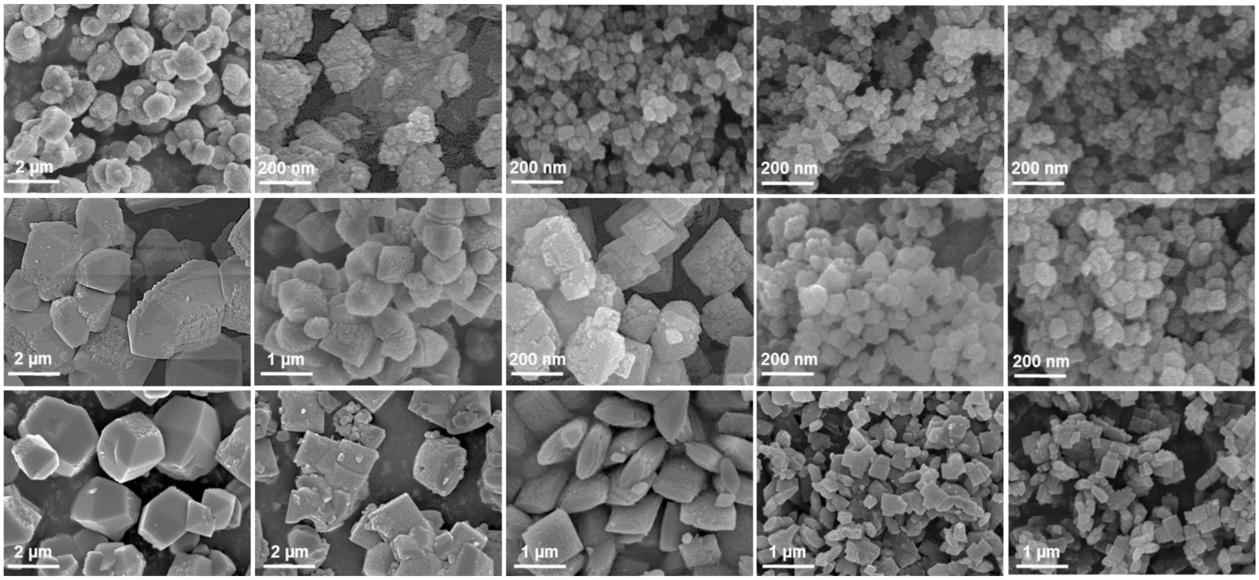

Fig. S8 FE-SEM images of as-made beta zeolites synthesized at the same mixture Si/Al ratio (12.5, top; 80, middle; 200, bottom) but different mixture HF/OSDA ratios (from left to right: 1.00, 0.75, 0.50, 0.25 and 0.13).

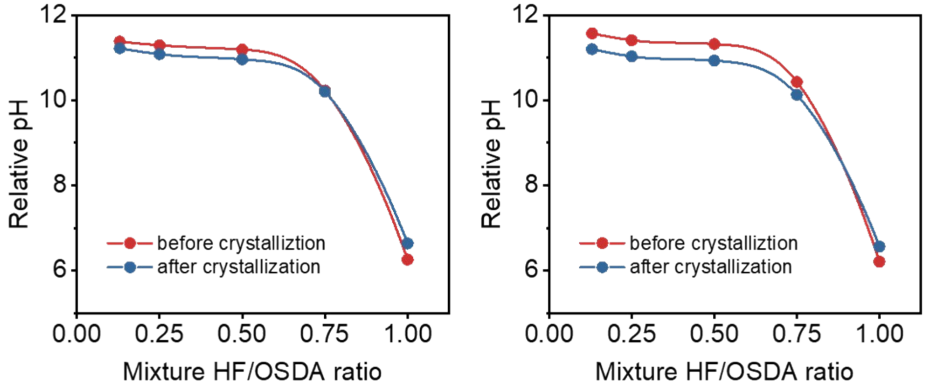

Fig. S9 Relative pH value as a function of HF/OSDA ratio of starting mixtures with Si/Al ratios of 80 (left) and 200 (right) before and after crystallization of beta zeolites. The pretreatment conditions of starting mixtures and final products before pH measurements are the same as those in Fig. S3.

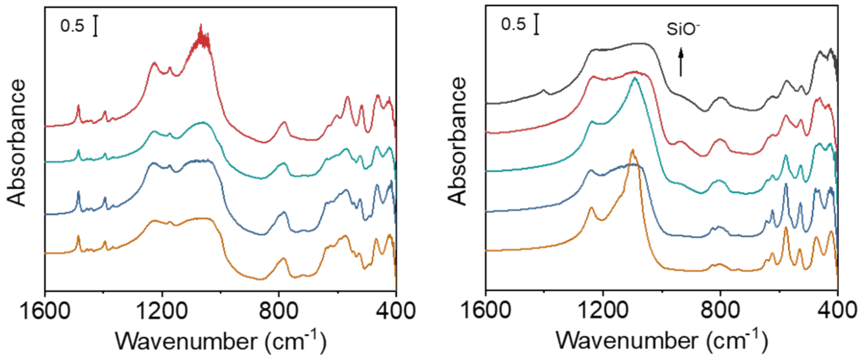

Fig. S10 IR spectra in the framework vibration region of the as-made (left) and proton forms (right) of beta zeolites synthesized at the same mixture HF/TEAOH ratio (0.50) but different Si/Al ratios (from top to bottom: 12.5, 40, 80 and 200). The spectrum of the proton form of a commercial beta zeolite (black) with Si/Al = 12.5 is given in the right panel.

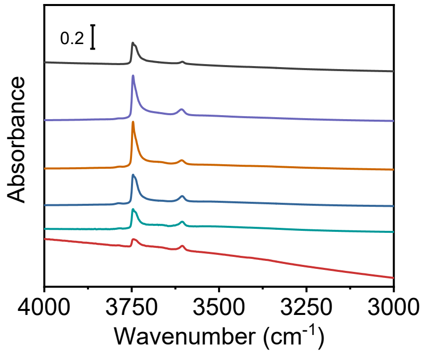

Fig. S11 IR spectra in the OH stretching region of the proton form of beta zeolites synthesized at the same mixture Si/Al ratio (40) but different HF/OSDA ratios (from bottom to top: 0.13, 0.25, 0.50, 0.75 and 1.00). Also given is he spectrum of the proton form of a commercial beta zeolite (black) with Si/Al = 12.5.

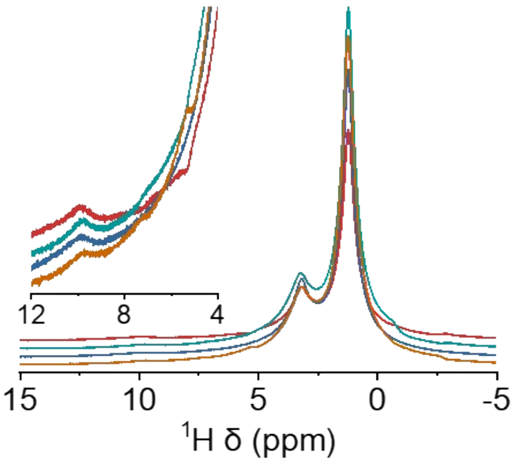

Fig. S12 ^{1}H MAS NMR spectra of as-made beta zeolites synthesized at the same mixture HF/TEAOH ratio (0.50) but different Si/Al ratios (from top to bottom: 12.5, 40, 80 and 200).

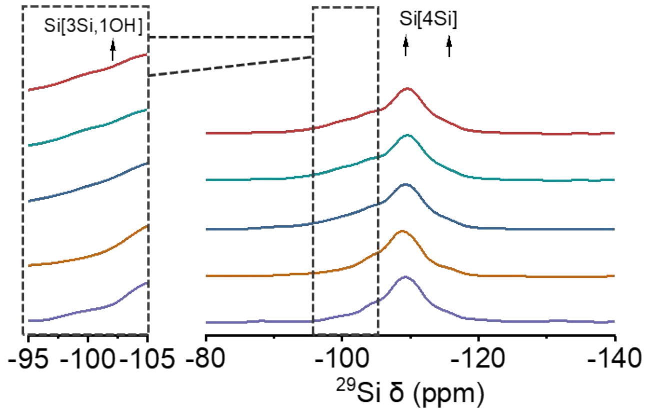

Fig. S13 ^{1}H-^{29}Si CP MAS NMR spectra of the as-made beta zeolites obtained using mixtures with the same Si/Al ratio (40) but different HF/OSDA ratios (from bottom to top: 0.13, 0.25, 0.50, 0.75 and 1.00).

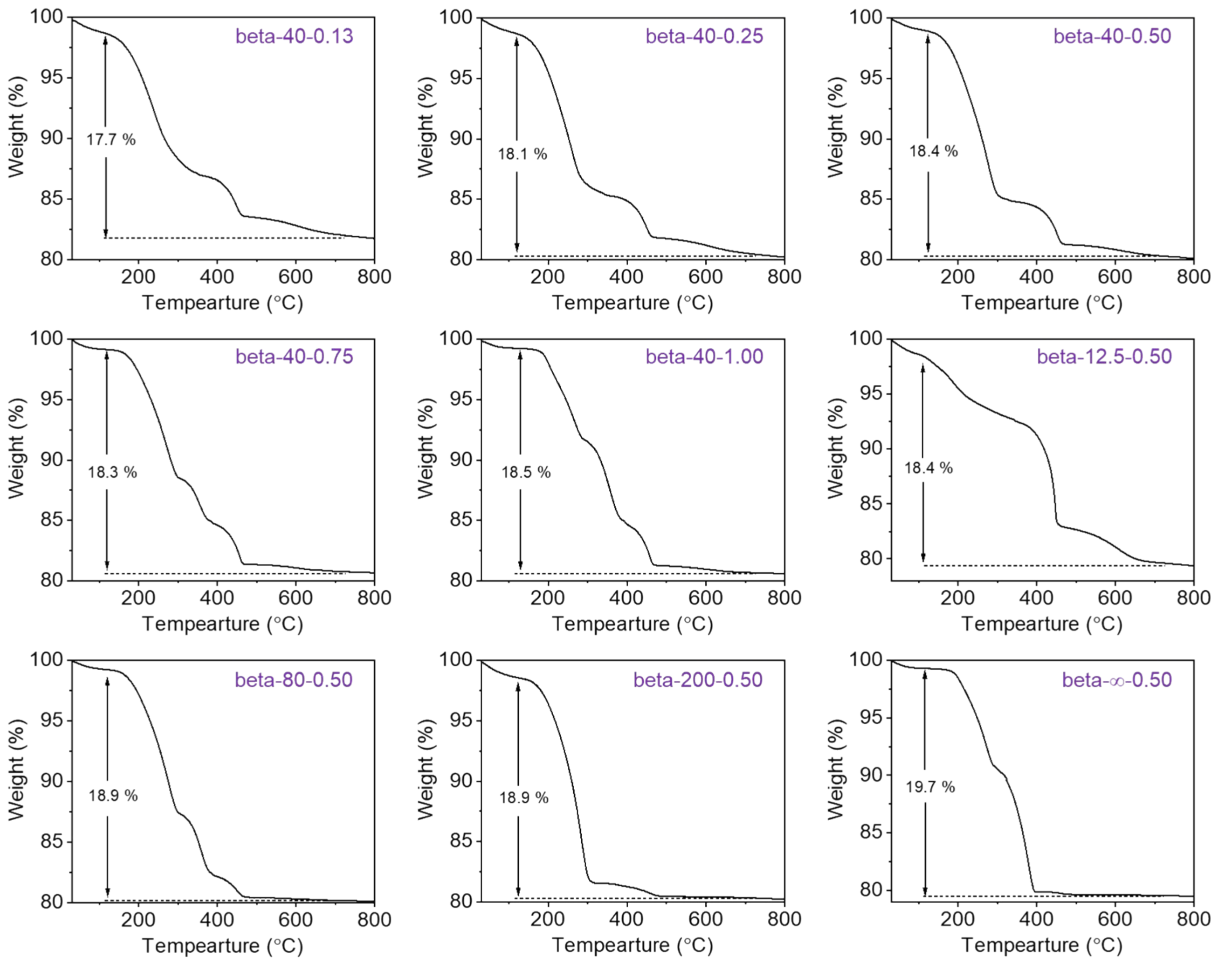

Fig. S14 TGA curves of as-made beta zeolites obtained using mixtures with different Si/Al ratios (12.5 – ∞) and HF/OSDA ratios (0.13 – 1.00).

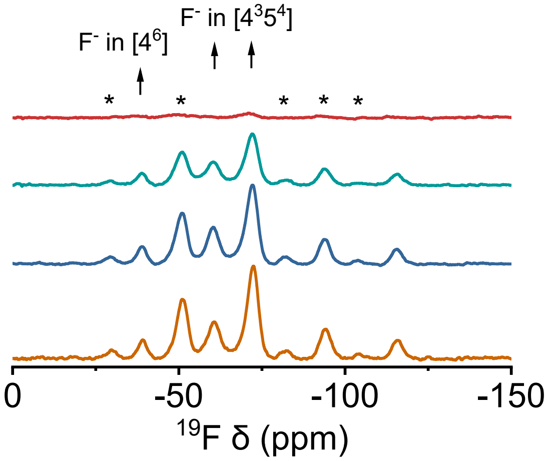

Fig. S15 ^{19}F MAS NMR spectra of as-made beta zeolites synthesized at the same mixture HF/TEAOH ratio (0.50) but different Si/Al ratios (from top to bottom: 12.5, 40, 80 and 200). The spinning sidebands are indicated by asterisks.

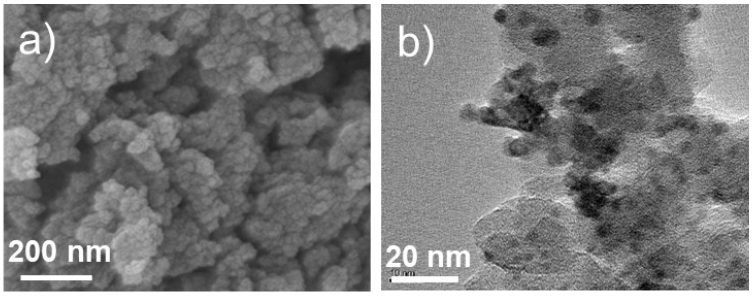

Fig. S16 (a) FE-SEM image of the NH_4^+-form of a commercial beta zeolite with Si/Al = 12.5 (NH_4-beta(c)-12.5) and (b) TEM image of 3.0Pd/H-beta(c)-12.5.

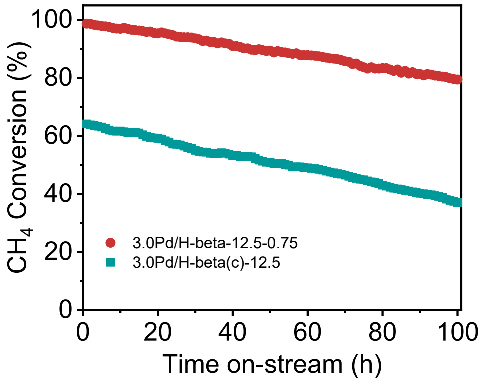

Fig. S17 CH_4 conversion at 375 °C as a function of time on-stream over 3.0Pd/H-beta(c)-12.5 and 3.0Pd/H-beta-12.5-0.75 catalysts. Reactant feed composition: 1500 ppm CH_4, 5% O_2 and 10% H_2O balanced with N_2 at a GHSV of 100,000 h^{-1}.

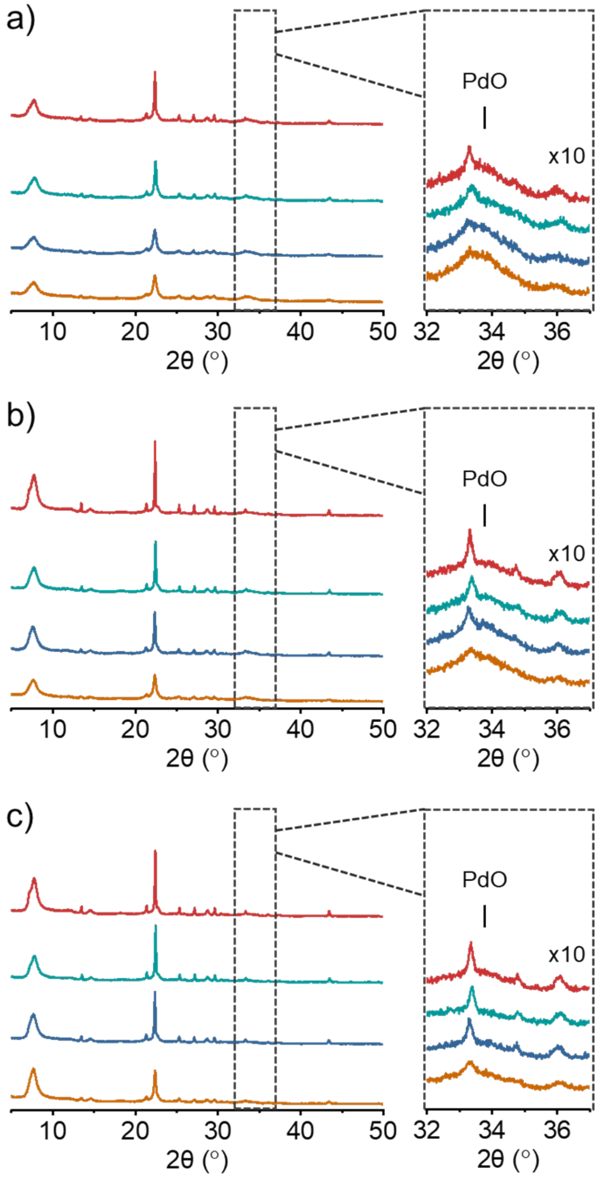

Fig. S18 PXRD patterns of a series of (a) 3.0Pd/H-beta-12.5-*n*, (b) 3.0Pd/H-beta-40-*n* and (c) 1.5Pd/H-beta-80-*n* catalysts, where *n* is the HF/OSDA ratio of synthesis mixtures used for beta zeolite crystallization and is, from bottom to top, 0.13, 0.25, 0.50, 0.75 and 1.00.

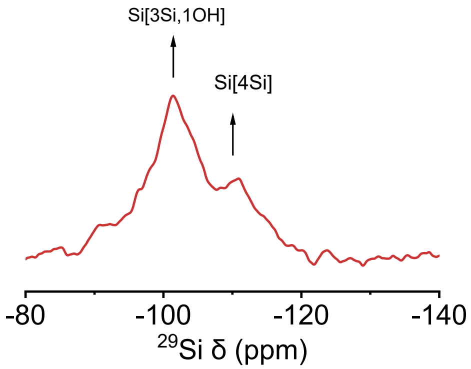

Fig. S19 ^1H-^{29}Si CP MAS NMR spectra of the proton form of the commercial beta zeolite with Si/Al = 12.5.

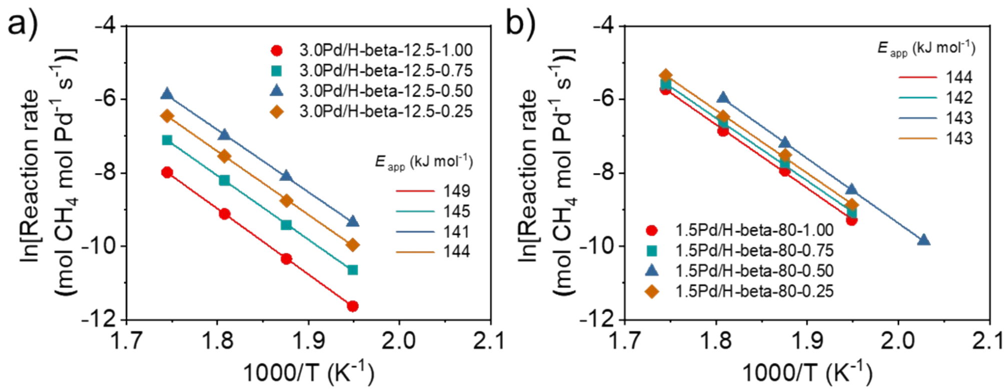

Fig. S20 Arrhenius plots and apparent activation energies for wet CH_4 combustion over a series of (a) 3.0Pd/H-beta-12.5-*n* and (b) 1.5Pd/H-beta-80-*n* catalysts, where *n* is the HF/OSDA ratio of synthesis mixtures used for beta zeolite crystallization.

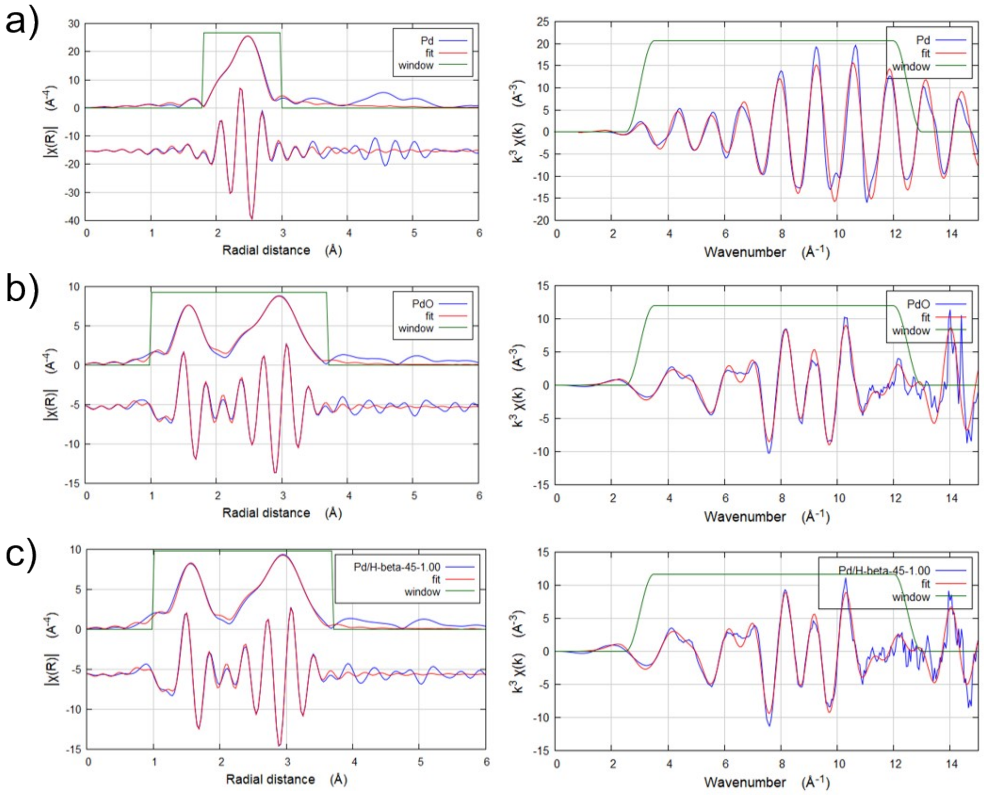

Fig. S21 Curve-fitting results of the Pd *K*-edge EXAFS spectra in *R*-space (left) and *k*-space (right): (a) Pd foil, (b) PdO and (c) 3.0Pd/H-beta-40-1.00.

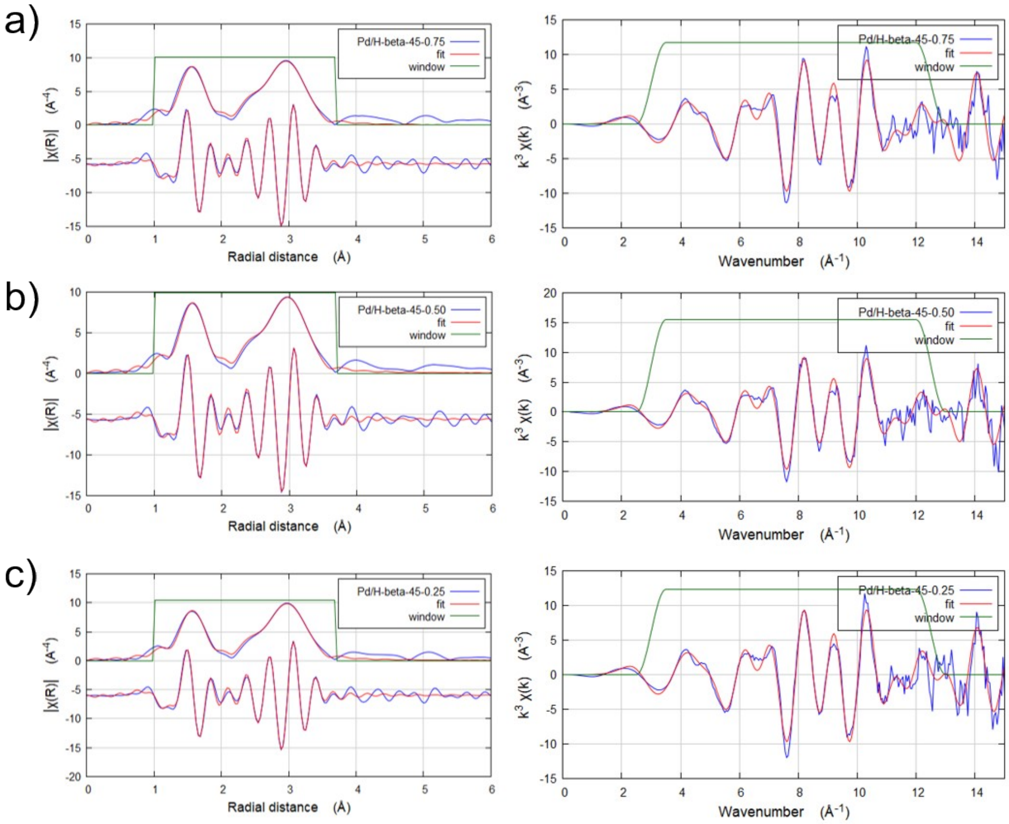

Fig. S22 Curve-fitting results of the Pd *K*-edge EXAFS spectra in *R*-space (left) and *k*-space (right): (a) 3.0Pd/H-beta-40-0.75, (b) 3.0Pd/H-beta-40-0.50 and (c) 3.0Pd/H-beta-40-0.25.

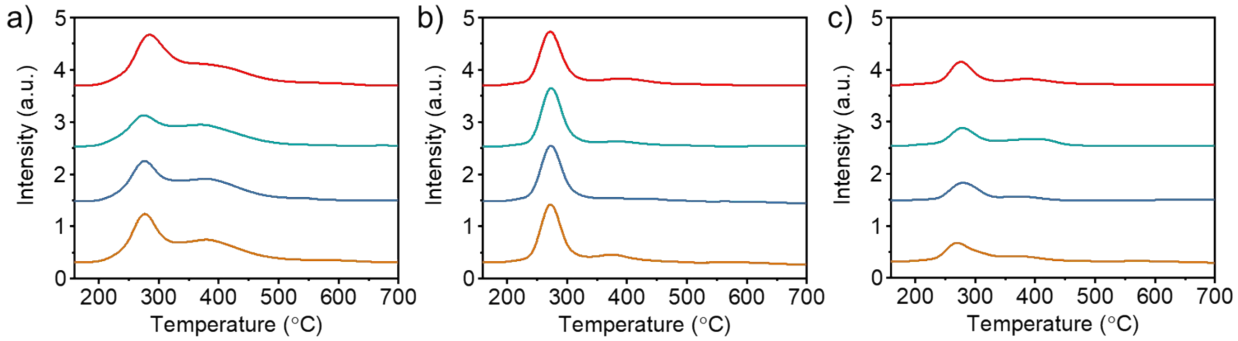

Fig. S23 NH_3 TPD profiles of a series of (a) 3.0Pd/H-beta-12.5-*n*, (b) 3.0Pd/H-beta-40-*n* and (c) 3.0Pd/H-beta-80-*n* catalysts, where *n* is the HF/OSDA ratio of synthesis mixtures used for beta zeolite crystallization and is, from bottom to top, 0.25, 0.50, 0.75 and 1.00.

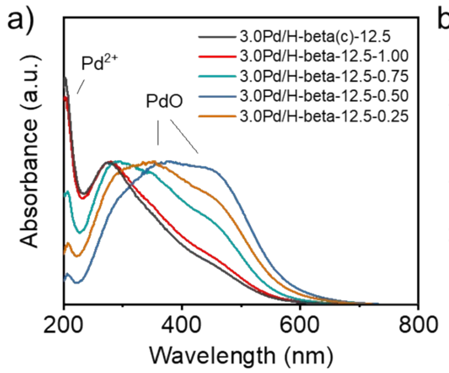

Fig. S24 UV-vis DR spectra of a series of (a) 3.0Pd/H-beta-12.5-*n* and (b) 1.5Pd/H-beta-80-*n* catalysts, where *n* is the HF/OSDA ratio of synthesis mixtures used for beta zeolite crystallization.

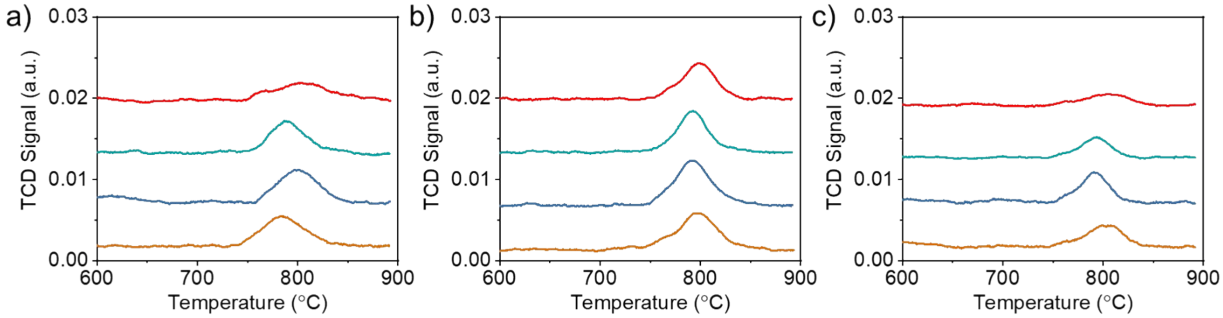

Fig. S25 O_2 TPD profiles of a series of (a) 3.0Pd/H-beta-12.5-*n*, (b) 3.0Pd /H-beta-40-*n* and (c) 1.5Pd /H-beta-80-*n* catalysts, where *n* is the HF/OSDA ratio of synthesis mixtures used for beta zeolite crystallization and is, from bottom to top, 0.25, 0.50, 0.75 and 1.00.

Table S1 Relative peak intensities of the ^{29}Si MAS NMR spectra of as-made beta zeolites synthesized in this study

	Relative intensity (%)	
Sample ID[a]	$Peak_{-104\ ppm}$	$Peak_{-109\ ppm}$ + $Peak_{-115\ ppm}$
beta-40-0.13	20.3	79.7
beta-40-0.25	19.6	80.4
beta-40-0.50	18.0	82.2
beta-40-0.75	16.9	83.1
beta-40-1.00	15.8	84.2

[a] The last two numbers in the sample ID are the Si/Al and HF/OSDA ratios of the synthesis mixture used in zeolite crystallization, respectively.

Table S2 Physical properties of the representative Pd/beta catalysts

Catalyst ID	Pd[a] (wt%)	Na[a] (wt%)	Pd^{2+} exchange level[a] (%)
3.0Pd/H-beta-12.5-1.00	3.0		64
3.0Pd/H-beta-12.5-0.75	2.9		60
3.0Pd/H-beta-12.5-0.50	3.0		62
3.0Pd/H-beta-12.5-0.25	2.9		58
3.0Pd/H-beta(c)-12.5	3.1		56
3.0Pd/H-beta-40-1.00	2.8		165
3.0Pd/H-beta-40-0.75	3.1		170
3.0Pd/H-beta-40-0.50	3.1		173
3.0Pd/Na-beta-40-0.50	3.1	0.6	173 (80)[b]
3.0Pd/H-beta-40-0.25	3.0		172
1.5Pd/H-beta-80-1.00	1.5		170
1.5Pd/H-beta-80-0.75	1.6		175
1.5Pd/H-beta-80-0.50	1.5		173
1.5Pd/H-beta-80-0.25	1.6		176

[a] Determined by elemental analysis. Ion exchange level = $n \times (M^{n+}/Al) \times 100\%$ (i.e. Pd^{2+}, $n = 2$; Na^{+}, $n = 1$). [b] The value in parentheses is the Na^{+} exchange level.

Table S3 Curve-fitting results of Pd *K*-edge EXAFS data for the representative Pd/beta catalysts

Catalyst ID	Path	CN[a]	R^b (Å)	$\sigma^{2,c}$ (× 10^3 Å^2)	$\Delta E_0{}^d$ (eV)	*R*-factor[e]
Pd foil	Pd-Pd	12	2.74	4.8	-1.8	0.003
	Pd-O	4	2.01	0.3		
PdO	Pd(-O-)Pd1	4	3.05	3.1	-3.1	0.006
	Pd(-O-)Pd2	8	3.44	4.9		
	Pd-O	4.6	2.02	1.6		
3.0Pd/H-beta-40-1.00	Pd(-O-)Pd1	6.0	3.06	5.5	-1.0	0.007
	Pd(-O-)Pd2	6.0	3.45	4.6		
	Pd-O	4.5	2.02	1.0		
3.0Pd/H-beta-40-0.75	Pd(-O-)Pd1	5.8	3.06	5.2	-0.3	0.006
	Pd(-O-)Pd2	6.2	6.45	4.5		
	Pd-O	4.5	2.02	1.3		
3.0Pd/H-beta-40-0.50	Pd(-O-)Pd1	6.0	3.06	5.6	-0.3	0.007
	Pd(-O-)Pd2	6.1	3.44	4.4		
	Pd-O	4.6	2.02	1.5		
3.0Pd/H-beta-40-0.25	Pd(-O-)Pd1	6.1	3.02	5.8	0.1	0.008
	Pd(-O-)Pd2	6.2	3.44	4.3		

[a] Coordination number. [b] Distance between the absorber and backscatter atoms. [c] Debye-Waller factor. [d] Energy shift of the Pd *K*-edge (24,350.0 eV). [e] Goodness of the fit value.
